# Computational design of serine hydrolases

**DOI:** 10.1126/science.adu2454

**Published:** 2025-04-18

**Authors:** Anna Lauko, Samuel J. Pellock, Ivan Anischenko, Kiera H. Sumida, David Juergens, Woody Ahern, Jihun Jeung, Alex Shida, Andrew Hunt, Indrek Kalvet, Christoffer Norn, Ian R. Humphreys, Cooper Jamieson, Rohith Krishna, Yakov Kipnis, Alex Kang, Evans Brackenbrough, Asim K. Bera, Banumathi Sankaran, K. N. Houk, David Baker

**Affiliations:** 1 Department of Biochemistry, University of Washington, Seattle, WA, USA; 2 Institute for Protein Design, University of Washington, Seattle, WA, USA; 3 Graduate Program in Biological Physics, Structure and Design, University of Washington, Seattle, WA, USA; 4 Graduate Program in Molecular Engineering, University of Washington, Seattle, WA, USA; 5 Department of Chemistry, University of Washington, Seattle, WA, USA; 6 Howard Hughes Medical Institute, University of Washington, Seattle, WA, USA; 7 Paul G. Allen School of Computer Science and Engineering, University of Washington, Seattle, WA, USA.; 8 Department of Chemistry and Biochemistry, University of California, Los Angeles, California, USA

## Abstract

The design of enzymes with complex active sites that mediate multistep reactions remains an outstanding challenge. With serine hydrolases as a model system, we combined the generative capabilities of RFdiffusion with a new ensemble generation method to design and filter enzymes starting from minimal active sites. Experimental characterization revealed catalytic efficiencies (*k*_*cat*_/*K*_*m*_) up to 4.3×10^3^ M^−1^ s^−1^ and crystal structures that closely match the design models (Cα RMSDs < 1 Å). Selection for structural compatibility across the reaction coordinate enabled identification of new catalysts in low-throughput screens and yielded serine hydrolases in five different folds not previously observed in this enzyme family. Our de novo approach provides insight into the geometric basis of catalysis and a roadmap for designing enzymes that catalyze multistep transformations.

## Graphical Abstract

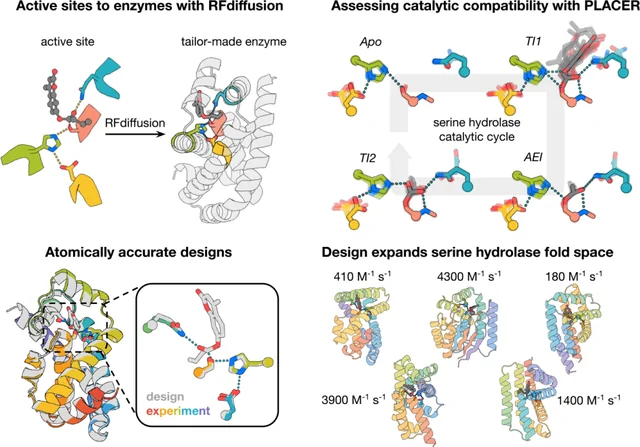

**Computational design of serine hydrolases.** RFdiffusion generates enzymes tailor-made for a given active site (top left). Assessing design compatibility across the catalytic cycle with the PLACER neural net (top right). Structural characterization of designs made with RFdiffusion and filtered with PLACER are atomically accurate (bottom left). Designed serine hydrolases expand the fold space of this ancient enzyme family (bottom right, catalytic efficiencies listed).

Enzymes are powerful catalysts that dramatically accelerate reaction rates in mild aqueous conditions. The ability to construct enzymes catalyzing arbitrary chemical reactions would have enormous utility across a wide range of applications, and hence, enzyme design has been a long-standing goal of computational protein design (1). De novo enzyme design has generally started from a specification of arrangements of catalytic residues around the reaction transition state (a theozyme), and sought to identify placements of this active site in pre-existing scaffolds (2–7). Utilizing fixed backbones restricts how accurately the catalytic geometry can be realized and has likely limited the activities of many designed enzymes to date prior to optimization by laboratory evolution, as recent studies of designed Kemp eliminases demonstrate (8–10). A further challenge of enzyme design is the preorganization of the active site such that the catalytic functional groups are accurately positioned relative to the transition state. Achieving preorganization is especially difficult for multistep reaction mechanisms, because the enzyme must preferentially stabilize multiple transition states and intermediates, and existing methods to evaluate design preorganization in silico are limited by low accuracy or computational cost (7, 11–14). To enable the accurate design of multistep enzymes, new methods are needed for both the generation of protein backbones tailored specifically to a given active site and assessment of their structural compatibility throughout the catalytic cycle.

Ester hydrolysis has served as a model reaction for computational enzyme design for decades (15–20), and justifiably so: numerous mechanisms catalyze ester hydrolysis, enabling a range of distinct design approaches to target this reaction, activity is easily monitored by absorbance and fluorescence with reporter substrates, and esterases are highly valuable in industrial processes, most recently for their application in plastic recycling (21–23). The textbook example of enzymatic ester hydrolysis is the double-displacement reaction mechanism employed by serine hydrolases, in which a serine nucleophile undergoes acylation to form a stable acyl-enzyme intermediate (AEI) that is subsequently hydrolyzed by an activated water. Despite extensive structural, mutational, and computational characterization of the mechanism of serine hydrolases found in nature (24–35), de novo design efforts attempting to employ this machinery have been unsuccessful, and to our knowledge, no previous efforts have successfully constructed a serine hydrolase that extends beyond the fold space found in nature. A major challenge in designing serine hydrolases is overcoming the stability of the AEI, the resolution of which is typically rate-limiting when activated esters are employed. Numerous previously designed enzymes and peptide-based systems inactivate or dramatically slow down after acylation (6, 15–17). In addition to this chemical challenge, constructing the serine hydrolase active site combines some of the most difficult current challenges in protein design: 1) the catalytic site is very complex, requiring the scaffolding of at least four individual residues with atomic precision, a task that state-of-the-art design tools struggle to achieve (36), 2) the serine nucleophile requires activation by construction of intricate hydrogen bond networks, and 3) the design must accommodate subtle conformational changes throughout the multistep catalytic cycle, and while there is recent progress in multistate design (37, 38), it remains challenging, particularly when the energetic difference between desired states are small.

Previous efforts to design esterases have circumvented the challenges presented by serine hydrolases by employing simpler, more easily designable active sites, leveraging nucleophiles more activated than serine, and by targeting reaction mechanisms that do not require the formation of stable covalent intermediates. For example, previously designed metallohydrolases skip the AEI by activating water to cleave esters in a single step (18, 39), the non-canonical amino acid *N*_*δ*_-methylhistidine has been employed to make the AEI less stable (17), and cysteine has been used in place of serine due to its greater nucleophilicity (6, 15). Structural analysis of the resulting cysteine esterases indicated key interactions between the cysteine nucleophile and histidine base of the desired dyad or triad were not formed (6, 15), suggesting that the inherent chemical reactivity of the residues employed, not their coordinated effort, may have been responsible for the observed steady-state rate enhancements. Even with these chemical interventions, the efficiency of the initial computational designs remain far below the range observed for natural enzymes.

One hypothesis for the lack of designed serine hydrolases to date is a potential geometric incompatibility between the complex hydrolase active site and the sets of fixed protein scaffold libraries previously employed (6). We investigated whether increasing scaffold diversity could help identify backbones that more accurately reconstruct the desired active site, and carried out a preliminary design campaign searching for placements of a serine hydrolase active site in a large library of scaffolds based on the Nuclear Transport Factor 2 (NTF2) fold (40) (fig. S1 and Computational methods, NTF2 design campaign). As in previous studies (7), experimental characterization of the resulting designs revealed activated serines but no catalytic turnover on ester substrates, despite a close match between the experimental and designed structures (fig. S2). We suspect that an inability to install key catalytic features into NTF2s, such as the backbone oxyanion hole contact common to all serine hydrolases, limited the function of these designs.

We reasoned that advances in deep learning for protein design could enable the design of proteins from scratch to directly scaffold the serine hydrolase active site and assess design compatibility for the entire multistep catalytic cycle. Recent advances in scaffolding functional sites with RFdiffusion have yielded improved *in silico* and experimental success rates across a range of design tasks (36, 41, 42); we aimed to use the same approach to generate serine hydrolases starting from geometric descriptions of an active site (Fig. 1A). To assess preorganization and functional interactions in each step of the catalytic cycle, we sought to leverage advances in deep learning-based prediction of protein-small molecule complexes by modeling structural ensembles of catalytic intermediates (Fig. 1B).

## Assessing reaction path compatibility with PLACER

**Figure 1.**
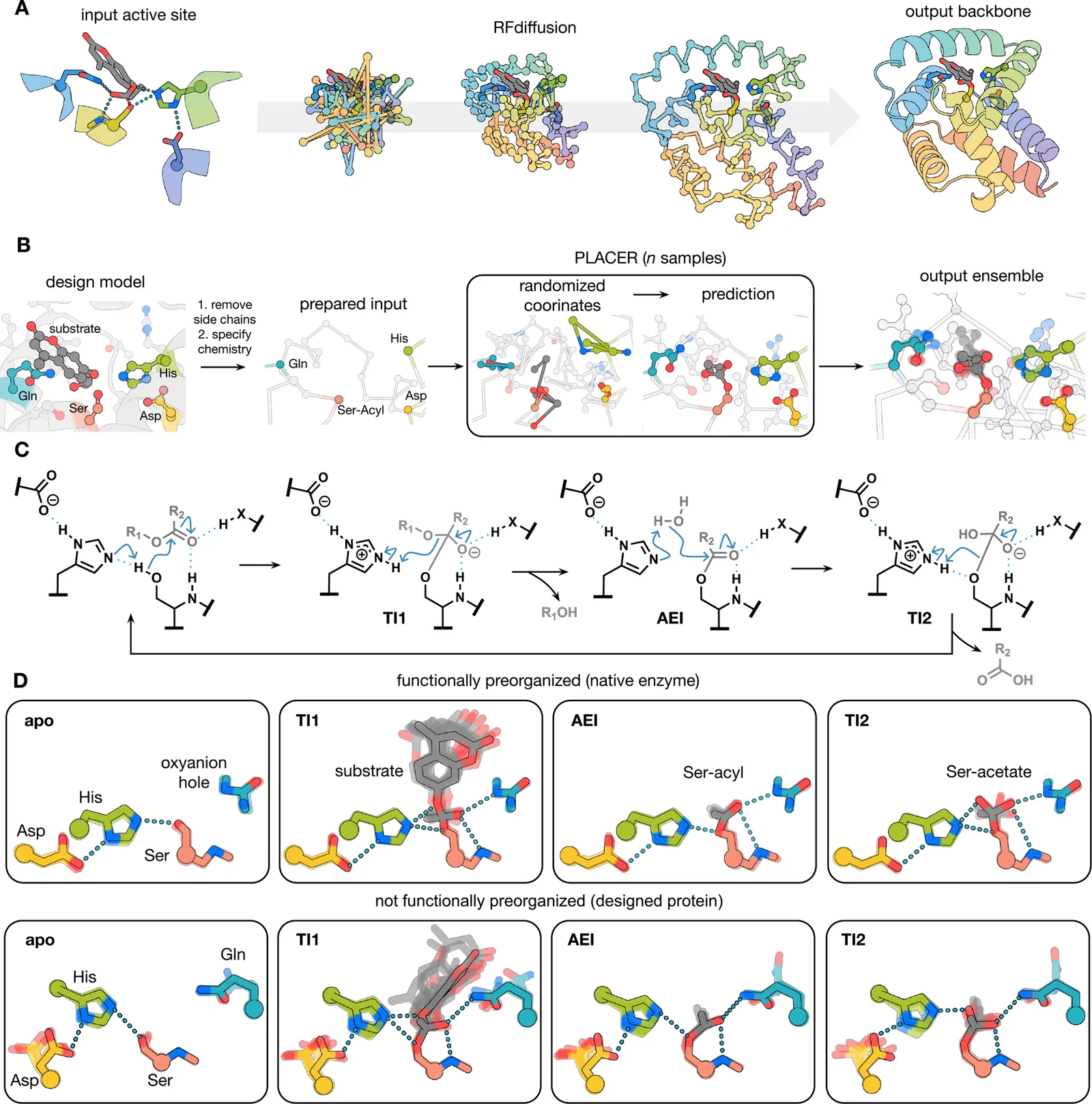
Design methods. (**A**) Active site specific backbone generation with RFdiffusion. Given the geometry of a possible active site configuration, RFdiffusion denoising trajectories generate backbone coordinates which scaffold the site. (**B**) Generation of active site ensembles with PLACER. The coordinates of the sidechains around the active site and any bound small molecule for the step in the reaction being considered are randomized, and *n* samples are carried out to generate an ensemble of predictions. (**C**) Mechanism of ester hydrolysis by serine hydrolases. (**D**) PLACER ensembles for distinct states along the reaction coordinate for hydrolysis of 4MU-Ac for a native serine hydrolase (top, PDB: 1IVY) and a designed serine hydrolase (bottom, **r3-josie**).

We set out to understand why previously designed serine hydrolases failed to appreciably catalyze ester hydrolysis and hypothesized that modeling each state of the reaction coordinate could be critical for assessing the ability of a design to achieve catalytic turnover. To model the extent to which a designed enzyme can form each of the key states along the reaction coordinate and to assess the preorganization of the active site residues in the desired catalytic geometries, we developed a deep neural network that, given 1) the backbone coordinates of a small molecule binding pocket or active site, 2) the identities of the amino acid residues at each position, and 3) the chemical structures of bound small molecules (but not their positions), generates the full atomic coordinates of the binding site, comprising both protein sidechains and small molecules. We trained this network, called PLACER (43), on protein-small molecule complexes in the PDB by randomizing the atomic coordinates of sidechains and small molecules within spherical regions with up to 600 heavy atoms, and seeking to minimize a loss function assessing the recapitulation of the atomic coordinates within the region. In benchmark tests, PLACER predicted regions within native structures with an average RMSD of 1.1 Å. PLACER is stochastic, and repeated runs from different random seeds yield an ensemble of models for the predicted region (Fig. 1B).

We used PLACER to generate structural ensembles for each step of the catalytic cycle for a set of native and previously designed serine hydrolases. The catalytic cycle of serine hydrolases can be divided into four steps (Fig. 1C). First, the substrate binds to the apoenzyme (apo) and the catalytic serine, deprotonated by the catalytic histidine, attacks the carbonyl carbon of the ester to form the first tetrahedral intermediate (TI1). Second, the catalytic histidine protonates the leaving group oxygen promoting its departure, leaving the active site serine covalently linked to the acyl group of the substrate (acyl-enzyme intermediate, AEI). Third, the histidine deprotonates a water molecule, which attacks the AEI to generate a second tetrahedral intermediate (TI2). Finally, this intermediate is resolved by histidine-mediated protonation of serine and release of the acyl group, reconstituting the free enzyme and completing the catalytic cycle. Throughout, negatively charged transition states and intermediates are stabilized by at least two hydrogen bond donors that constitute the oxyanion hole. Perturbation of the histidine pK_a_, which tunes its acid/base function, is mediated by interaction with aspartate or glutamate, the final residue in the triad (44–46).

Modeling this catalytic cycle with PLACER showed that native serine hydrolases are more preorganized than previous designed systems (Fig. 1D, fig. S2). At each step in the reaction coordinate, the catalytic residues sample the key hydrogen bonds essential for catalysis more often in native than designed serine hydrolases (fig. S3). Since the reaction rate should be proportional to the fraction of the enzyme in the active state, limited preorganization of the designed active sites is expected to compromise catalysis. To quantify the extent of active site formation in PLACER ensembles, we compute the frequency of formation of key interactions between the catalytic functional groups and reaction intermediates over each step of the reaction (see Computational methods, filtering section), and use this metric to assess new designs in the following sections.

## Design and characterization of serine hydrolases

**Figure 2.**
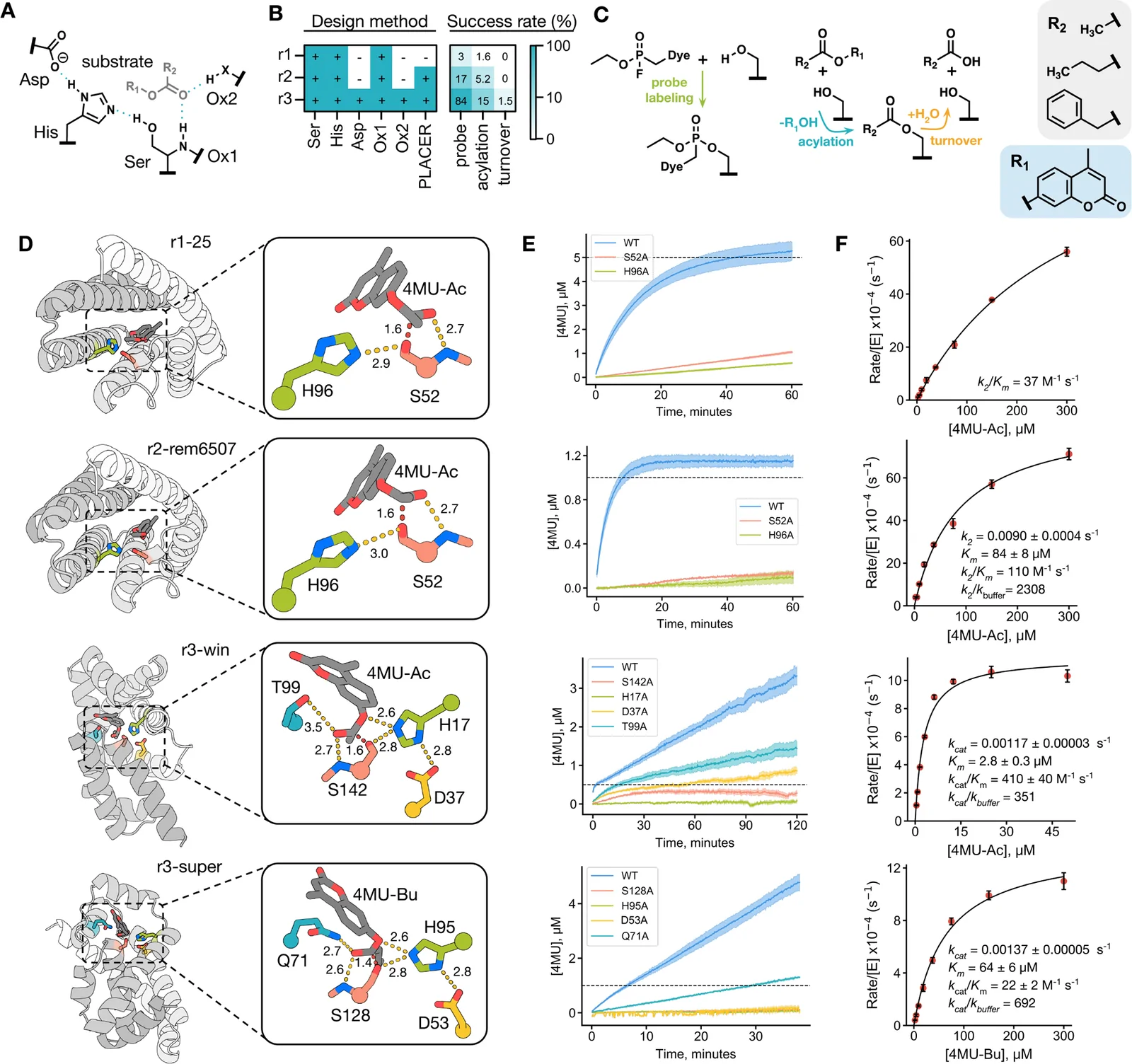
Functional characterization of designed serine hydrolases. (**A**) Chemical schematic of a serine hydrolase active site. (**B**) Summary of design method and experimental success rate for probe labeling, single turnover acylation, and catalytic turnover for each design round. (**C**) Chemical schematic depicting probe labeling, acylation, and catalytic turnover. (**D**) Fold (left) and active site (right) of serine hydrolase design models. (**E**) Reaction progress curves for the parent design and catalytic residue knockouts. Dashed line represents the enzyme concentration and shaded areas represent standard deviation of three technical replicates. (**F**) Michaelis-Menten plots derived from initial (r1, r2) or steady state velocities (r3). Error bars represent standard deviation of three technical replicates.

We next set out to design proteins with active sites of increasing complexity, using RFdiffusion to scaffold serine hydrolase active site motifs and PLACER to assess their preorganization in each step of the reaction (Fig. 2A,B). We designed catalysts for the hydrolysis of 4-methylumbelliferone (4MU) esters (Fig. 2C) that fluoresce upon hydrolysis. To generate active site motifs, we placed the catalytic sidechains around a QM-optimized transition state (see Computational methods) based on an analysis of natural hydrolases (33). To generate backbones to scaffold the catalytic machinery, we started from the backbone N, Cα, and C atoms of the key catalytic residues and their immediate neighbors (i.e. a contiguous three-residue segment) and used RFdiffusion to build up backbones starting from random noise that have coordinates that nearly exactly match the input motif (average all-atom RMSD ~ 0.1 Å) and form a binding pocket for the substrate (see Computational Methods, motif generation and backbone generation). To drive folding to the designed state, and to make favorable interactions with the substrate and active site residues, LigandMPNN (47) was used to design the sequence. Rosetta FastRelax (48) was used to refine the protein backbone and ligand pose, and the sequence was again designed with LigandMPNN with the new backbone as input (49). Following three cycles of LigandMPNN and FastRelax, the structures of the designs were predicted with AlphaFold2 (AF2) (50), and designs for which all catalytic residue Cα atoms were positioned within 1.0 Å of the design models were selected for experimental characterization (50) (see Computational methods, sequence design for details).

In the first two rounds of design, we built relatively simple active sites consisting of Ser-His dyads with a single oxyanion hole contact from the backbone amide of the serine (Fig. 2A,B), and explicitly evaluated the utility of PLACER to select designs for experimental characterization; round 1 designs were filtered with AF2 alone, while round 2 designs that passed the AF2 filter were selected for experimental screening if PLACER ensembles of the apo state indicated the key Ser-His hydrogen bond was formed (see Computational Methods, filtering). Only 1.6% of round 2 designs that passed the AF2 RMSD filter were predicted to be preorganized by PLACER. For experimental testing, we obtained synthetic genes encoding 129 and 192 designs for rounds 1 and 2, respectively, for *E. coli* overexpression and screening.

We used a fluorophosphonate (FP) activity-based probe and fluorescent 4MU-acetate (4MU-Ac) and 4MU-butyrate (4MU-Bu) ester substrates to identify designs with activated serines and esterase activity, respectively (Fig. 2C). The fraction of designs labeled by the FP probe in *E. coli* lysate increased 5-fold from 3% to 17% from round 1 to round 2 (Fig. 2B and fig. S4). Designs that reacted with the FP probe were purified and incubated with 4MU esters, and two round 1 designs (1.6%) and 10 round 2 designs (5.2%) showed catalytic activity. Retrospective PLACER analysis of the round 1 designs revealed that the Ser-His H-bonds in the two catalytically active designs were predicted to be among the most preorganized (fig. S5). PLACER filtering of round 2 designs on the extent of formation of the key Ser-His H-bond not only increased the fraction of designs exhibiting FP probe labeling and enzymatic activity, but also resulted in higher activities (Fig. 2E,F). The progress curves for these round 1 and 2 designs plateau after approximately one enzyme equivalent of fluorescent product is formed (Fig. 2E), suggesting the serine acylates but that the resulting AEI fails to hydrolyze, the rate-limiting step in the cleavage of activated esters (32). When incubated with substrate, mass spectra of these designs revealed a mass shift corresponding to acylation, further supporting protein inactivation following formation of the acylated intermediate (fig. S6).

We hypothesized that incorporating a histidine-stabilizing catalytic acid and a second oxyanion hole H-bond donor in a third round of designs (round 3) and filtering for PLACER preorganization in both the apo and AEI states could generate designs capable of catalytic turnover via hydrolysis of the AEI. For round 3 designs, we required all catalytic triad and oxyanion hole H-bonds to be highly preorganized in PLACER ensembles of both the apo and AEI states. Of 132 round 3 designs, 111 (84%) displayed FP probe labeling, 20 hydrolyzed 4MU substrates (18%), and two designs (1.5%) displayed multiple turnover activity (Fig. 2B,E). Active designs from all three rounds showed significantly reduced activity upon mutation of any one of the catalytic residues (Ser, His, Asp/Glu, and oxyanion sidechain contact) (Fig. 2E), suggesting that the observed activities are dependent on the designed active site. To determine the kinetic parameters of the active designs, initial or steady-state rates were measured to determine *k*_*2*_/*K*_*m*_ or *k*_*cat*_/*K*_*m*_ for single-turnover and multiple-turnover designs, respectively (Fig. 2F and fig. S7). For the two designs that displayed catalytic turnover, called **‘r3-super’** and **‘r3-win**,’ *k*_*cat*_/*K*_*m*_ values were 22 M^−1^ s^−1^ (*k*_*cat*_ = 0.00137 ± 0.00005 s^−1^, *K*_*m*_ = 64 ± 6 μM) and 410 M^−1^ s^−1^ (*k*_*cat*_ = 0.00117 ± 0.00003 s^−1^, *K*_*m*_ = 2.8 ± 0.3 μM), respectively for the more preferred of the two 4MU substrates (**r3-win** and **r3-super** preferentially hydrolyzed 4MU-Ac and 4MU-Bu, respectively (fig. S8)). Despite the low *K*_*m*_ observed for **r3-win**, we were unable to reach saturation of the initial burst phase of the reaction by increasing substrate concentration up to 100 μM (fig. S9), suggesting that *K*_*s*_ >> *K*_*m*_ and that the low apparent *K*_*m*_ observed for **r3-win** is a result of rapid acylation and not tight substrate binding.

## Structural characterization of designed serine hydrolases

**Figure 3.**
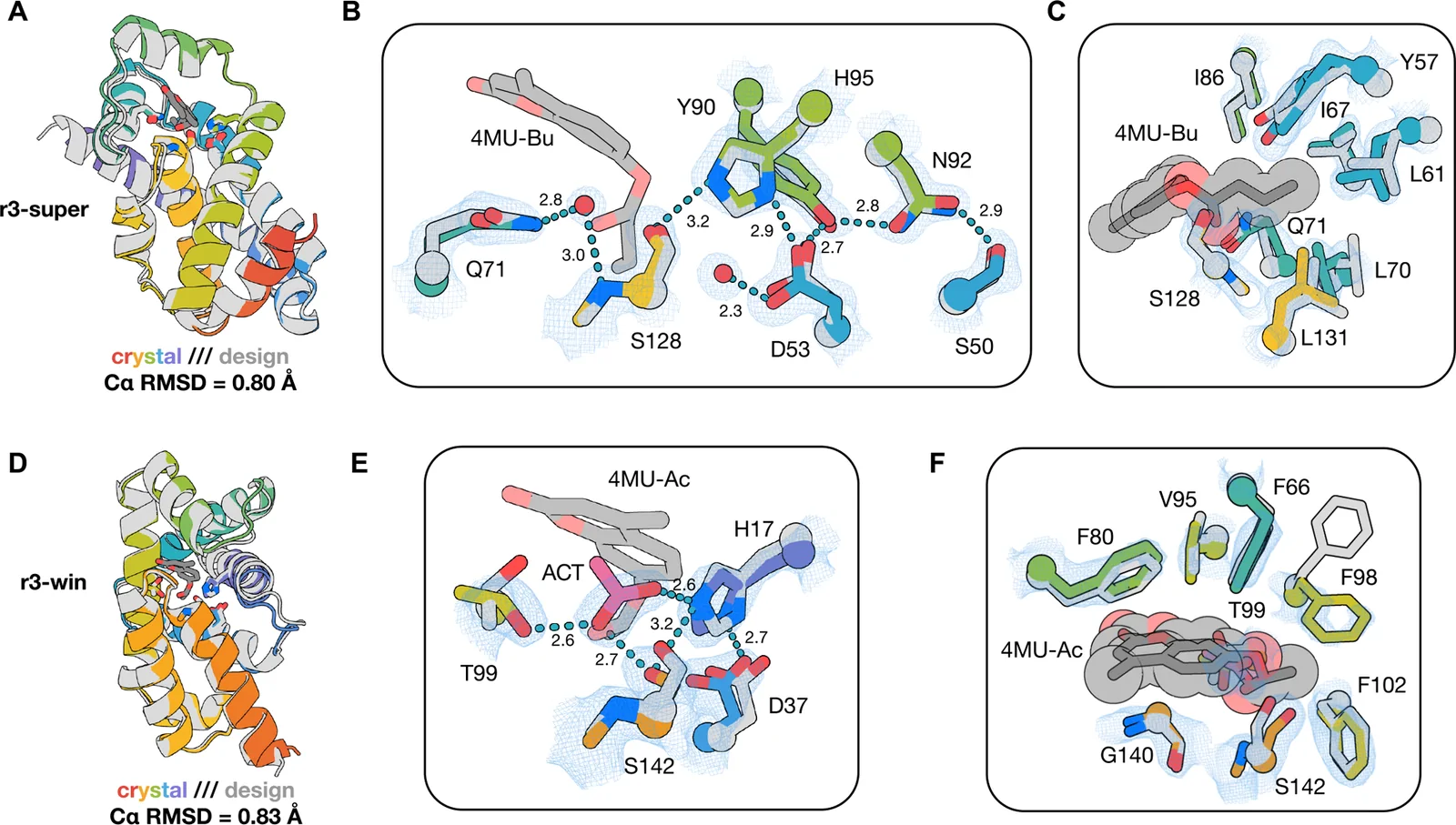
Structural characterization of designed serine hydrolases. (**A, D**) Structural superposition of design models (gray) and crystal structures (rainbow) for **r3-super** (**A**) and **r3-win** (**D**). (**B** and **E**) Active site overlays of design models (gray) and crystal structures (rainbow) of **r3-super** (**B**) and **r3-win** (**E**) with 2Fo-Fc map shown at 1σ (blue mesh). (**C** and **F**) Superposition of substrate binding sites of the design models (gray) and crystal structures (rainbow) of **r3-super** (**C**) and **r3-win** (**F**) with 2Fo-Fc map shown at 1 σ (blue mesh). Distances shown in Å.

We pursued x-ray crystallography to determine the accuracy with which **r3-super** and **r3-win** were designed. We were able to solve crystal structures of both **r3-super** and **r3-win**, and found that they had very low Cα RMSDs of 0.8 Å οver 165 residues and 0.83 Å over 160 residues (Fig. 3A,D), respectively, to the design models. The design accuracy extends to the geometry of the active site: the sidechain conformations of the catalytic residues are in atomic agreement for **r3-super** (all-atom RMSD = 0.38 Å over 22 atoms) and for **r3-win** (all-atom RMSD = 0.86 Å over 20 atoms) except for a rotamer shift in the sidechain oxyanion contact, Thr99 (Fig. 3B,E). In the active site of **r3-super**, a water molecule sits above the nucleophilic serine and forms hydrogen bonds with the oxyanion hole contacts, which likely mimics the positioning of the carbonyl oxygen of its ester substrate (Fig. 3B). Similarly, in **r3-win**, an acetate molecule is positioned at the catalytic center and hydrogen bonds to the catalytic serine (Ser142), the sidechain oxyanion hole (Thr99), and the histidine acid/base residue (His17) (Fig. 3E).

While the structures were solved in the absence of bound small molecule substrate or transition state analogue, overlay of the design model and crystal structure of **r3-super** reveals high shape complementarity to the butyrate acyl group of its preferred substrate (Fig. 3C and S8). At the same time, the 4MU moiety is largely exposed, corroborating the selectivity of **r3-super** for 4MU-Bu over 4MU-Ac and suggesting that substrate binding, in this case, is largely driven by binding to the acyl group. For **r3-win**, a rotamer shift in F98 in the crystal structure would clash with the butyrate moiety, and indeed, **r3-win** is selective for the smaller substrate 4MU-Ac that avoids this clash (Fig. 3F and fig. S8).

The structures of **r3-super** and **r3-win** are very different from known structures; the closest matches found from Foldseek searches against all available databases have TM-scores of 0.52 and 0.46 for **r3-super** and **r3-win**, respectively (at or below the 0.5 cutoff below which structures are considered to have different topological folds), are proteins of unknown function, and have no similarity to known hydrolases at the fold or active site level (fig. S10A,B), demonstrating that the design method employed here yields structural solutions for serine hydrolase activity that extend well beyond those found in nature, expanding the structural space of this ancient enzyme family.

## Filtering for preorganization across the reaction coordinate improves catalysis

**Figure 4.**
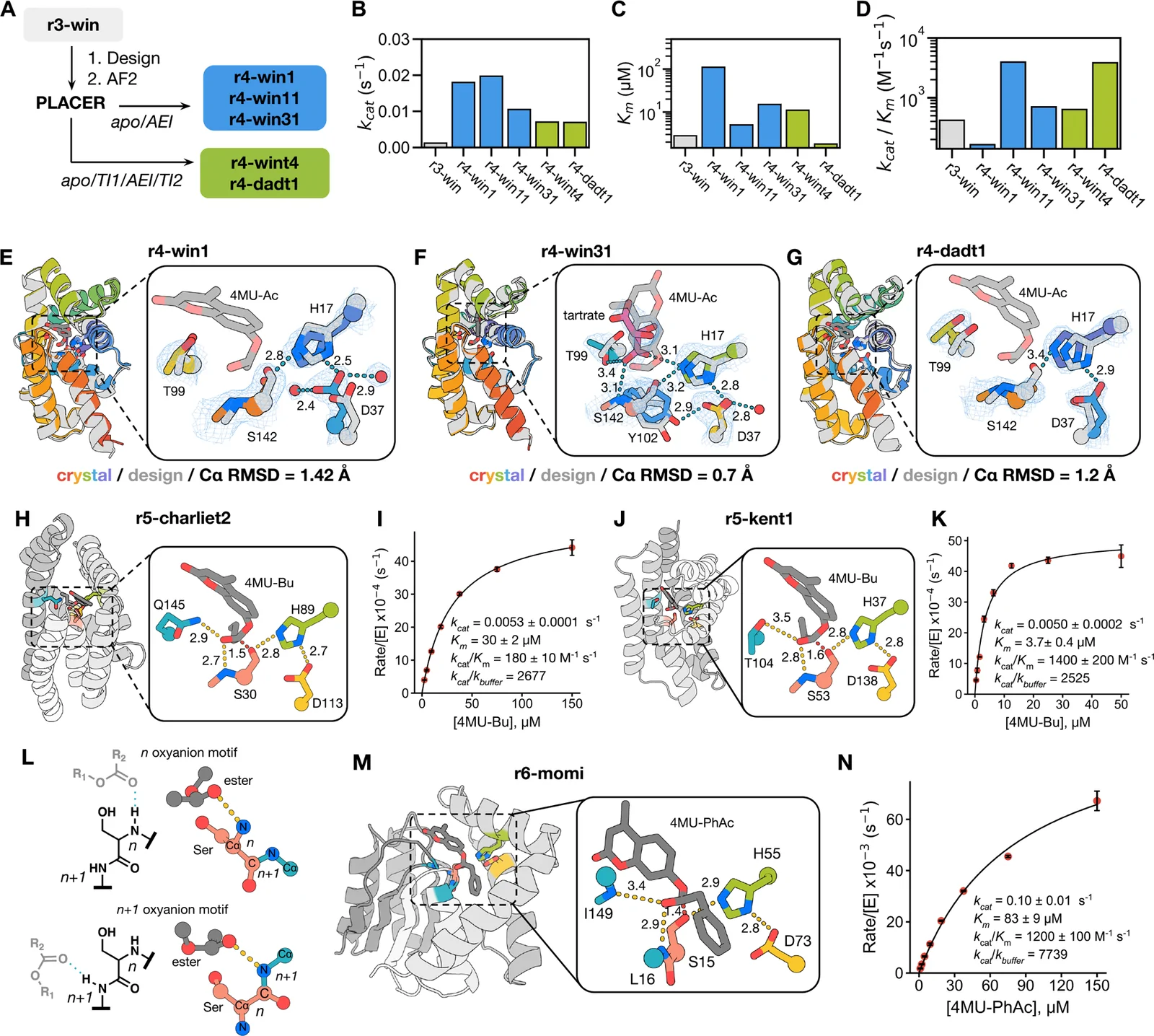
Computational redesign and more complex folds improve catalysis. (**A**) Computational pipeline for redesign of **r3-win**. (**B,C,D**) *k*_*cat*_ (**B**), *K*_*m*_ (**C**), and *k*_*cat*_/*K*_*m*_ (**D**) of parent **r3-win** compared to computational redesigns. (**E,F,G**) Structural superposition of design model and crystal structure of **r4-win1** (**E**), **r4-win31** (**F**), and (**G**) **r4-dadt1** with 2Fo-Fc map shown at 1σ. (**H,I,J,K**) Design models (**H,J**) and Michaelis-Menten plots (**I,K**) for active designs with distinct folds. (**L**) Chemical and structural comparison of *n* and *n*+*1* oxyanion hole motifs. (**M**) Design model of **r6-momi** that utilizes two backbone amide oxyanion hole contacts, one from an *n*+*1* backbone amide. (**N**) Michaelis-Menten plot for **r6-momi** with 4MU-PhAc. Error bars represent standard deviation of three technical replicates.

We next sought to generate and compare designs filtered explicitly with PLACER for preorganization over two states (apo and AEI) or over all four states of the reaction path by carrying out additional iterations of LigandMPNN and FastRelax starting from the active design **r3-win** (fixing only the identities of the four catalytic residues) (Fig. 4A and fig. S1). We obtained genes encoding 45 two-state filtered designs for experimental characterization, all of which were diverse in sequence compared to the original designs (mean sequence identity to the parent design of 58% and 61% within the active site), and found 38 (84%) labeled with FP-probe (fig. S11A), and 9 (20%) displayed activity over background in a lysate screen (fig. S11C). Three of these, **r4-win1**, **r4-win11**, and **r4-win31**, displayed higher *k*_cat_ values compared to the starting design: **r3-win** has a *k*_cat_ of 0.00117 s^−1^, which increases 15-fold in **r4-win1** (0.018 s^−1^), 17-fold in **r4-win11** (0.0197 s^−1^), and 9-fold in **r4-win31** (0.0105 s^−1^) (Fig. 4B and fig. S7). Of the 11 four-state filtered designs tested, 10 (91%) labeled with FP-probe (fig. S11B) and 8 (73%) displayed activity (fig. S11D). Two of these, **r4-dadt1** and **r4-wint4**, displayed higher catalytic efficiencies than **r3-win**, with *k*_cat_/*K*_m_ values of 3800 M^−1^ s^−1^ and 640 M^−1^ s^−1^, driven by increases to *k*_*cat*_ and decreases in *K*_*m*_ relative to **r3-win** (Fig. 4B,C and fig. S7). Catalytic triad residue knockouts for all designs showed significant reductions in activity. In **r4-win11** and **r4-win31**, mutation of stabilizing residues in the second shell of the active site that H-bond to the catalytic aspartate also significantly reduced activity (fig. S12). The two redesigns with the highest *k*_*cat*_ values (**r4-win1** and **r4-win11**) do not display burst phase kinetics, suggesting that deacylation is no longer rate-limiting (fig. S7).

We determined the crystal structures of **r4-win1**, **r4-win31**, and **r4-dadt1** and comparison to the design models revealed Cα RMSDs of 1.42 Å, 0.7 Å, and 1.2 Å, respectively (Fig. 4E,F,G). For **r4-win1**, the active site closely matches the designed architecture (mean all-atom RMSD = 0.54 Å) (Fig. 4E), and T99, the oxyanion hole contact, occupies the designed rotamer, which may account for the 15-fold increase in *k*_*cat*_ compared to **r3-win**, in which T99 is rotated relative to the designed rotamer (Fig. 3E). In chain B of the **r4-win1** structure, the catalytic serine partially occupies a flipped conformer with an occupancy of 0.23 (fig. S13). For **r4-win31**, five chains are present in the asymmetric unit, all of which closely match the design model (average Cα RMSD = 0.7 Å) at the backbone level (Fig. 4F and S13). Analysis of the active site across all chains in the asymmetric unit revealed mobility in the catalytic serine, sidechain oxyanion threonine, and a second shell tyrosine (fig. S13), but overall a very close match to the design model active site with a mean all-atom RMSD of 0.7 Å. Tartrate, derived from the crystallization solution, fit the electron density present in the active site of all five chains, and forms hydrogen bonds with the serine, histidine, and oxyanion hole contacts (Fig. 4F), likely mimicking key contacts employed throughout the catalytic cycle. For **r4-dad_t1**, the active site closely matches the design model with a mean all-atom RMSD of 0.95 Å, and the T99 sidechain oxyanion residue occupies the designed conformation.

We next explored whether stringent PLACER filtering for optimal catalytic geometry and preorganization across the reaction coordinate could generate active esterases with novel backbone topologies and active site geometries. We performed sequence design and PLACER filtering for the complete reaction coordinate on round 3 backbones excluding **r3-win** (fig. S1), and of 20 designs tested, two (**r5-charliet2** and **r5-kent1**) displayed significant esterase activity, with catalytic efficiencies of 180 M^−1^ s^−1^ and 1400 M^−1^ s^−1^ (Fig. 4H,I,J,K), suggesting that structural variability in intermediate states of the reaction coordinate may have limited otherwise functional designs. We also used sequence design combined with PLACER filtering to modify the substrate selectivity of **r4-win1**, converting it from accepting only the small acyl group of 4MU-Ac to processing larger substrates such as 4MU-phenylacetate (4MU-PhAc) (fig. S14).

To test the generality of RFdiffusion combined with PLACER filtering, we applied it to a different active site configuration in which the oxyanion hole consists of two backbone amides, rather than a backbone amide and a sidechain H-bond donor, and where the first backbone amide of the oxyanion hole is the residue following the catalytic serine (*N*+*1*) rather than the catalytic serine itself (*N*) as in the previous designs (Fig. 4L). We used the RFdiffusion and LigandMPNN/FastRelax design pipeline to generate 66 designs for this new catalytic site and the larger 4MU-PhAc substrate (fig. S1). The most active of these, **r6-momi**, displayed a *k*_*cat*_/*K*_m_ of 1240 M^−1^ s^−1^ and *k*_*cat*_ of 0.1 s^−1^, a 5-fold faster rate than **r4-win11**, the previous best design in terms of turnover number. The distribution of folds generated by RFdiffusion for this active site geometry differed from that of the original geometry, with more α/β fold solutions (as in the case of **r6-momi**), showing how the RFdiffusion buildup approach crafts overall protein structure topology to the specific active site of interest. Like **r6-momi**, natural esterases to our knowledge exclusively employ the *N*+*1* oxyanion hole motif, suggesting that it is uniquely suited for ester hydrolysis. The high activity achieved without any prior experimental characterization for this new catalytic site shows that filtering for preorganization across the reaction cycle can yield novel catalysts in one shot.

Several experimental results identify areas to address for improved function. First, **r5-kent1** inactivates after roughly 10 turnovers, and mass spectra of the catalyst and the serine knockout incubated with substrate reveal stable acylated species (fig. S15), indicating that designs that hydrolyze the AEI are still susceptible to inactivation, potentially from off-mechanism acylation events in the active site or acylation-induced conformational changes. Second, mutation of the sidechain oxyanion hole residue had variable effects on activity. In three designs (**r4-dadt1**, **r5-charliet2**, **r5-kent1**) from design rounds 4 and 5 that underwent stringent PLACER filtering, mutation of the sidechain oxyanion hole residue had a modest effect on activity, suggesting limited contribution to catalysis (fig. S12). Analysis of the oxyanion hole geometries in these designs and others in earlier design rounds reveal in-plane hydrogen bonds to the oxygen of the substrate carbonyl (fig. S16, Supplementary Text), in opposition to the ones found in nature, which are perpendicular to the plane of the carbonyl, a geometry thought to selectively stabilize the transition state and likely destabilize the ground state (34, 51, 52).

We next explored whether existing designs could be improved by rebuilding suboptimal regions using RFdiffusion. Using the **r6-momi** backbone as input to RFdiffusion, we built out the N-terminus to further stabilize the active site but made no changes to the parent backbone or sequence (fig. S1 and fig. S17). Of 65 designs tested, all showed activity, and one design, **r7-momi120**, displayed a catalytic efficiency of 4300 M^−1^ s^−1^, 3.5-fold greater than **r6-momi**, driven by a 2-fold increase in *k*_*cat*_ and 2-fold decrease in *K*_*m*_ (fig. S17). We also used RFdiffusion to improve the suboptimal in-plane (with respect to the substrate carbonyl) oxyanion hole H-bond formed by Gln71 in **r3-super**. The serine protease subtilisin utilizes a chemically similar sidechain oxyanion hole, Asn155, positioned instead in an out-of-plane orientation (fig. S16A). Using the subtilisin oxyanion hole geometry as a guide, we mutated Gln71 to Asn in **r3-super**, and repositioned it to form an analogous out-of-plane H-bond to the substrate carbonyl, then rebuilt the surrounding backbone of the protein with RFdiffusion to accommodate this change (fig. S18). Of the 150 designs screened, the two most active designs, **r8-superfast** and **r8-supercool**, showed 8-fold and 7-fold improvements in *k*_*cat*_ over the parent design **r3-super** (*k*_*cat*_ = 0.00137 s^−1^), and 19-fold and 13-fold improvements in *k*_*cat*_/*K*_*m*_, respectively (fig. S18). These results highlight productive design interventions made possible by RFdiffusion that are not easily accessible with traditional engineering tools like rational mutagenesis and directed evolution, where the sequence can be readily changed but not easily augmented with new structural features.

## Structural determinants of catalysis

The high structural conservation of catalytic geometry in native serine hydrolases suggests that it is close to optimal for catalysis (33, 53), but it is difficult to assess how activity depends on the detailed geometry of the interactions of the transition states with the catalytic serine, histidine, and oxyanion hole functional groups since while the identities of the catalytic residues can be readily changed by mutation, it is not straightforward to systematically vary backbone geometry. In contrast, our de novo buildup approach samples a wide range of catalytic geometries. To investigate how active site geometry and preorganization influence catalytic activity, we generated PLACER ensembles of all 812 experimentally characterized designs, categorized as inactive, FP probe labeling, acylation, and catalytic turnover, for each reaction step in the hydrolysis of 4MU-Ac (including design rounds 1–3 and previous NTF2-based designs). The following features were associated with activity.

**Figure 5.**
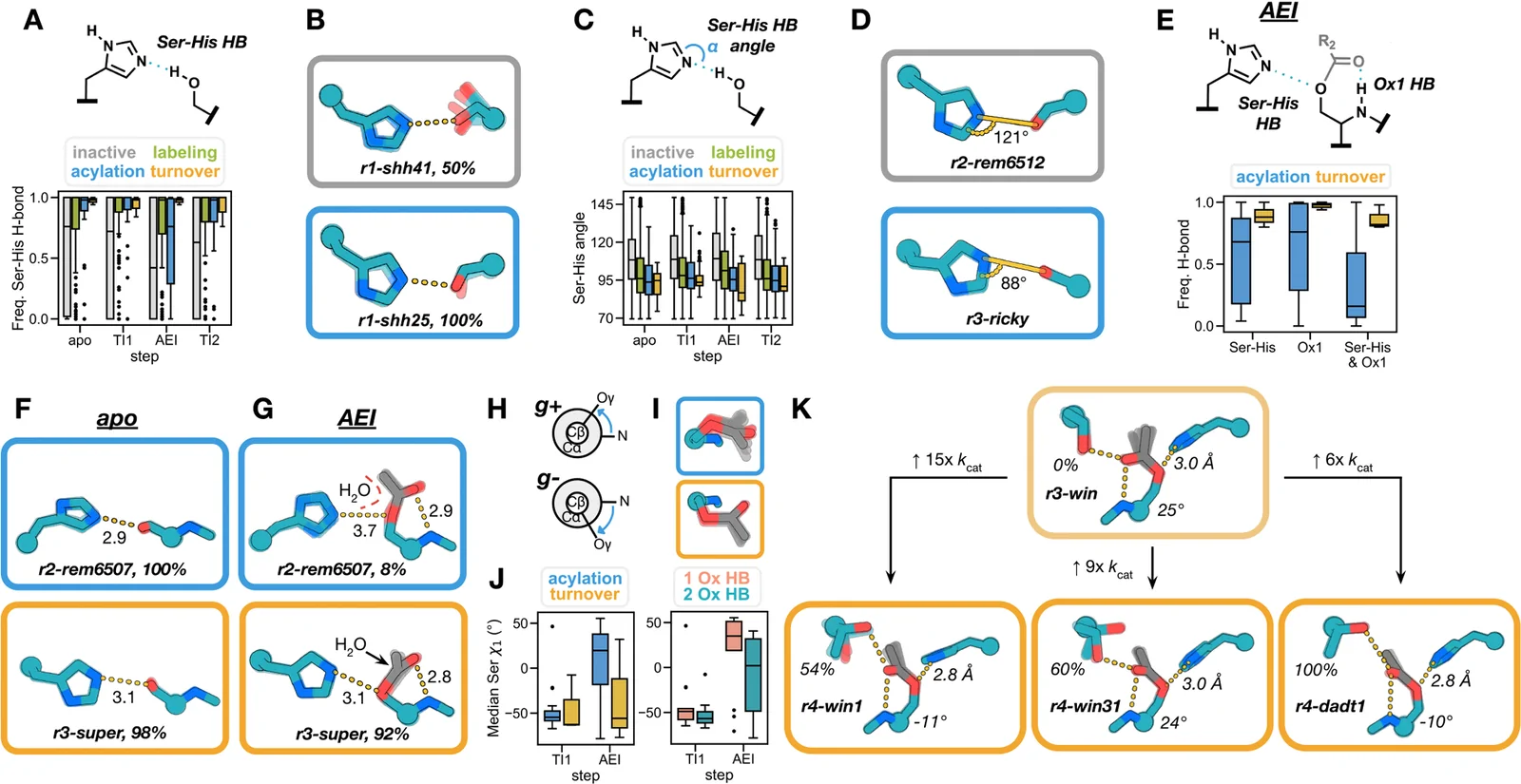
PLACER ensembles reveal geometric determinants of catalysis. (**A**) Frequencies of catalytic Ser-His H-bond formation in PLACER ensembles for each reaction step, grouped by experimental outcome. (**B**) Apo PLACER ensembles of representative inactive (top) and acylating (bottom) designs. (**C**) Median angle (*α*) between serine Oγ, histidine Nϵ and Cϵ across PLACER ensembles of inactive and acylating designs. (**D**) Apo PLACER ensembles of representative inactive (top) and acylating (bottom) designs, angle indicates median *α*. (**E**) AEI PLACER ensemble H-bond frequencies for designs that undergo acylation or full turnover. (**F**) PLACER ensembles of the apo state for an acylating (top) and multiple turnover design (bottom). (**G**) PLACER ensembles of the AEI state for a representative design that undergoes acylation (top) and a design that catalyzes turnover (bottom). Measurements shown represent median distances (Å) of key H-bonds indicated for each ensemble and percentages represent frequency of H-bond formation across all PLACER trajectories. (**H**) Newman projections of serine *g*+ and *g*− rotameric states (left). (**I**) PLACER ensembles of an acylating design (top) and a design that catalyzes turnover (bottom). (**J**) Median serine *χ*_*1*_ angle across TI1 and AEI state PLACER ensembles for designs that catalyze acylation or turnover (left) and for the same designs grouped by number of oxyanion hole H-bonds. (**K**) AEI state PLACER ensembles for **r3-win**, **r4-win1**, **r4-win31**, and **r4-dadt1**, with percent of frames with correct oxyanion hole rotamer, Ser *χ*_*1*_ angle, and catalytic Ser-His H-bond distance shown. Boxplots represent median, upper and lower quartiles; whiskers extend 1.5×IQR above and below the upper and lower quartiles (respectively). Observations falling outside these ranges plotted as outliers.

Increased preorganization and bending of the Ser-His H-bond were associated with higher rates of probe-labeling, acylation, and turnover. All designs capable of catalyzing turnover displayed highly preorganized Ser-His H-bonds across all four states, while inactive designs often displayed rotamer shifts causing loss of the interaction (Fig. 5A,B). Designs that catalyzed turnover had Ser(Oγ):His(Nε-Cε) bond angles that were more acute (median, all states = 94°) than inactive designs (median, all states = 108°), which were more similar to serine-histidine hydrogen bonds across the PDB (~125°) (34) (Fig. 5C). This acute H-bond is consistent with the reaction mechanism, as this geometry allows histidine to participate, without changing conformation, in all of the necessary proton transfers involving serine, the leaving group oxygen in TI1, and the hydrolytic water (35, 54). This compromise in positioning is observed not only in our active designs but also in many of those found in nature (34, 54, 55).

The geometry of the serine rotamer throughout the catalytic cycle was also strongly correlated with experimental outcome. For designs that display acylation or turnover, we found that serine largely occupies the active *g*− rotamer (53) in the apo state. Designs that display turnover retain the *g*− serine conformer upon formation of the AEI, but designs that irreversibly acylate switch to the *g*+ rotamer in the AEI (Fig. 5H,I,J). The *g*+ serine rotamer is catalytically incompetent in these designs because it leads to an acyl group conformation that occludes interaction of the hydrolytic water with histidine (Fig. 5G), increases the median Ser-His H-bond distance (Fig. 5G), and reduces the frequency that the Ser-His and oxyanion hole-acyl group H-bonds form (Fig. 5E). The same retention of the *g*− rotamer in the AEI is observed in native crystal structures (35). PLACER analysis also revealed that the presence of a second oxyanion hole residue favors the active *g*− serine rotamer: those designs with only one oxyanion hole H-bond (from the backbone amide of the serine nucleophile) shift from *g*− to *g*+ upon acylation, while designs with two oxyanion hole H-bonds predominantly occupy *g*− Ser rotamers (Fig. 5J, right). The second oxyanion hole contact in serine hydrolases thus not only stabilizes the transition state but likely helps orient intermediates in catalytically productive conformations.

Differential preorganization may also explain activity trends in the **r3-win**, **r4-win1**, **r4-win31**, and **r4-dadt1** series. PLACER analysis of the crystal structures of these designs revealed that in the AEI state, the more active redesigns **r4-win1**, **r4-win31**, and **r4-dadt1** sample the designed T99 oxyanion hole rotamer in 56, 60, and 100% of predictions, respectively, while **r3-win** never adopts this rotamer (Fig. 5K). Although both observed rotamers place T99 Oγ within hydrogen bonding distance of the oxyanion, the designed rotamer-oxyanion dihedral angle (91°) adopted by the redesigns much more closely matches the angles observed in native serine hydrolases, suggesting it is likely more optimal for selective transition state stabilization (34, 51, 52). We also observed differences in the serine rotameric state and the preorganization of the acyl group in the AEI state. Both **r3-win** and **r4-win31** occupy the catalytically unfavorable *g*+ rotamer across the entire AEI ensemble, while **r4-win1** and **r4-dadt1** both display a less pronounced rotameric shift, which leads to shorter Ser-His H-bond distances (mean 2.8 Å in **r4-win1** and **r4-dadt1** compared to 3.1 Å in **r3-win** and **r4-win31**). Overall, the acyl groups of **r4-win1** and especially **r4-win31** and **r4-dadt1** display significantly less conformational heterogeneity than that of **r3-win**, which may increase the likelihood of histidine-mediated water attack (Fig. 5K).

## Discussion

The substantial catalytic efficiencies, the complexity of the active sites, and the atomic accuracy of the designs described here represent major advances in computational enzyme design. The serine catalytic triad plus oxyanion hole mechanism involves complex machinery that is challenging to scaffold (compared to, for example, the Kemp eliminase, which requires only a general base in a hydrophobic environment (2)), necessitates chemical activation of serine, and proceeds through a complex multistep mechanism that traverses a chemically stable AEI. The designs herein are novel serine hydrolases in this ancient enzyme family and have efficiencies up to 4300 M^−1^ s^−1^, a significant improvement in function for computationally designed enzymes. For example, the previously designed esterase OE1 has a *k*_*cat*_/*K*_*m*_ = 210 M^−1^ s^−1^ and only reached a comparable efficiency (3190 M^−1^ s^−1^) to the designs described here after four rounds of directed evolution and screening over 12,000 clones, despite the use of a more activated *N*_*δ*_-methylhistidine nucleophile (17). The closest comparable de novo design in terms of mechanism, in which a cysteine-based catalytic triad was mutated into a peptide-based helical barrel that proceeds via a more activated thioester intermediate (15), has a *k*_*cat*_/*K*_*m*_ of 3.7 M^−1^ s^−1^ and *k*_*cat*_ of 0.0005 s^−1^, 1000x and 400x less than the most efficient and highest turnover design (**r7-momi120**) characterized in this study. The ability to accelerate the hydrolysis of a chemically stable acyl-enzyme intermediate has been a decades-old challenge in enzyme design. To approximate the deacylation rate enhancement achieved by our fastest design, we compare the uncatalyzed rate of hydrolysis of ethyl acetate (2.5–5.0)×10^−10^ s^−1^, (56)) to the lower limit of the deacylation rate constant of **r6-momi** (*k*_*cat*_, 0.076 s^−1^, pH 7.0, 25°C), yielding an estimated rate enhancement of over 10^8^. Taken together, the design of serine hydrolases spanning five folds not represented in natural esterases, considerable improvement in activity over previously designed esterases, and acceleration of deacylation represent key advances in enzyme design.

The designs described here are not as efficient as native serine hydrolases with their cognate substrates (e.g. the *k*_*cat*_/*K*_*m*_ of acetylcholinesterase with acetylcholine is >10^8^ M^−1^ s^−1^) (57), but they have activities comparable to natural proteases for activated esters (α-chymotrypsin with *p*-nitrophenyl acetate *k*_*cat*_/*K*_*m*_: 3530 M^−1^ s^−1^, *k*_*cat*_: 0.0053 s^−1^; subtilisin with *p*-nitrophenyl acetate *k*_*cat*_/*K*_*m*_: 610 M^−1^s^−1^, *k*_*cat*_: 0.23 s^−1^) (58, 59), and are within the distribution of efficiencies observed in nature (57). Higher *k*_*cat*_ could likely be achieved through optimization of the catalytic geometry, further preorganization of the active site (8, 9), and increasing active site complexity. Acetylcholinesterase employs three backbone amide hydrogen bonds to the oxyanion and an additional network of hydrogen bonds to stabilize the catalytic aspartate (60, 61). The current designs do not employ this machinery, and comparison of catalytic triad and oxyanion hole geometries to those found in highly efficient native serine hydrolases highlights differences that could be responsible for the remaining activity gap (see Supplementary Text) (34, 51, 52). Our de novo buildup approach using RFdiffusion coupled with PLACER ensemble analysis to ensure design accuracy and preorganization should allow us to test these hypotheses by direct construction, which should complement more traditional approaches based on structural examination, computational studies using QM/MM, and optimization by experimental approaches like directed evolution.

Previous efforts to design catalytic triad-based designs have failed to achieve multiple turnover; in some cases, such as our preliminary NTF2-based designs, a backbone amide oxyanion hole was impossible to achieve due to scaffold limitations, while in others based on native scaffolds, the histidine geometry was difficult to control which likely limited activation of the leaving groups and water (fig. S19) (7). De novo backbone generation building outward from a specified active site with RFdiffusion overcomes these limitations by enabling generation of almost any desired catalytic geometry. We further show that the deep neural network PLACER can rapidly generate ensembles for a series of reaction intermediates to predict preorganization, and provide insights that would otherwise require labor-intensive structural studies. For example, PLACER revealed pervasive off-target conformational changes in the acyl-enzyme intermediate, providing feedback on design flaws that would go unnoticed when considering only a single state in the catalytic cycle. The value of this approach is evident in the dramatic improvement in experimental success rate upon filtering with PLACER, suggesting that such ensemble generation will be useful for enzyme design moving forward. While the designs described here do utilize a known mechanism, the geometries sampled and the folds that scaffold them are distinct from those found in native proteins, and the insights provided by PLACER for these geometries suggests that the approach should prove valuable for assessing catalytic geometries for which no native precedent exists. We anticipate that the ability to precisely position multiple catalytic groups using RFdiffusion, and to assess active site organization throughout a complex reaction cycle using PLACER should enable the design of a wide variety of new catalysts, such as PETases, amidases, and ligases, in the near future.

## Materials and Methods

### NTF2 design campaign

Catalytic geometries from a previous analysis of native serine hydrolases (33) were used to generate constraint files for use in the RosettaMatch algorithm (62). The scaffold set used for matching was a set of idealized Nuclear Transport Factor 2 (NTF2) fold proteins generated with trRosetta (40). After matching, sequence design was performed using LigandMPNN and FastRelax and designs were filtered using AlphaFold2 as described below. An additional filter was used requiring that all catalytic hydrogen bonds in the active site be formed in the AlphaFold2 prediction.

### Computational design of serine hydrolases

#### Motif generation

Motifs were built in an iterative process. First, a substrate rotamer in a transition state geometry (either 4MU-Bu or 4MU-Ac) was placed in accordance with geometries in ref 33 in relation to a 3-residue stub of the serine and local oxyanion hole from one of two natural serine hydrolase crystal structures, in which all residues other than serine were mutated to alanine (*N* oxyanion hole: 1scn, residues 220–222; *N*+*1* oxyanion hole: 1lns, residues 347–349). The transition state geometry of the substrate ester group was determined by DFT geometry optimization (B3LYP-D3(BJ)/6–31G(d)). Next, positions and rotamers of histidine on 3-residue helical or strand stubs flanked by alanine were sampled around the catalytic serine and filtered for those structures in which the histidine simultaneously formed hydrogen bonds with the catalytic serine and the substrate leaving group oxygen. This process resulted in 108 unique motifs for design rounds 1 and 2. For the round 3 motifs, initially the aspartate or glutamate residue and second oxyanion hole hydrogen bond were added in a similar manner using geometric sampling of hydrogen-bonding conformations and rotamers. However, backbones produced from these motifs had exceedingly low AF2 success rates, presumably due to the generation of incompatible combinations of backbone conformations. To ensure that the remaining catalytic residue stubs were placed in physically plausible geometries, we generated 10,000 backbones with RFdiffusion using the simple substrate-Ser-His motifs as input, and then searched these backbones using Rosetta for positions on secondary structure that could accommodate the aspartate or glutamate triad residue to hydrogen bond to histidine. These stubs were then extracted, and in a final step, the same process was repeated to generate stubs for the second oxyanion hole, considering all hydrogen bond donating sidechains, ultimately producing 2238 unique round 3 motifs with Ser-His-Asp/Glu catalytic triads, and Ser/Thr/Tyr/His/Trp oxyanion holes.

#### Backbone generation

See supplemental methods for a detailed description of CA diffusion, which was employed to generate backbones to scaffold the generated active sites.

##### Backbone resampling for r6-momi and r3-super redesigns

The design model of **r6-momi** was provided as input to RFdiffusion and the entirety of the protein was fixed while a region of secondary structure was diffused at the N-terminus. The length of this region was randomly selected from a range of 20 to 50 amino acids at each of 1000 iterations of the diffusion task. The contigs flag for RFdiffusion was thus formatted as such: contigs:{region_length},A1–160. For each backbone, the sequence was kept fixed for the structure derived from the momi input. The sequence of the diffused region at the N-terminus was designed as described below. Ten sequences were generated per backbone.

To generate a **r3-super** input structure to RFdiffusion with an optimized oxyanion hole sidechain geometry, the active sites of **r3-super** and subtilisin (PDB: 1scn) were superimposed by alignment of the catalytic serine backbone atoms. The backbone region (residues 56–91) flanking the original oxyanion hole Gln71 was removed from **r3-super** and Asn155 from subtilisin was copied into the structure. RFam (63) was used to reconstruct the removed region while scaffolding the amide group of Asn with a new stretch of backbone with lengths between 48–58 residues. RFam is a new backbone generation model capable of scaffolding individual atoms or functional groups, simultaneously reasoning over the location of the scaffolded elements in sequence during the generation trajectory. 10,000 backbones were generated with this approach.

#### Sequence design

We performed three cycles of LigandMPNN (47) and Rosetta FastRelax (64) to design sequences for backbones generated from RFdiffusion. To encourage formation of hydrogen bond contacts to the catalytic histidine (for round 1 motifs) and to the catalytic aspartate/glutamate (round 3 motifs), the log probabilities used by LigandMPNN to select residues were biased toward polar amino acids for all residues with Cα within 8 Å of the active site. Catalytic residues were kept fixed and Rosetta enzyme constraints (62, 65) were applied during the relax steps to maintain the catalytic geometry during each LigandMPNN/FastRelax cycle. Constraints were defined for each hydrogen bonding interaction between the catalytic dyad, backbone oxyanion hole, and substrate using the starting motif geometry with tolerances of 0.1 Å for distances and 5° for angles and dihedrals. For designs with catalytic triads, the His-Asp interaction was constrained

#### Filtering

After sequence design, designs were filtered on the recapitulation of the motif catalytic geometry after FastRelax and the shape complementarity of the binding site to the substrate using Rosetta. Passing designs were used as input to AF2 (50) for single sequence structure prediction. AF2 was run using model 4 with three recycles. Designs were filtered for a global Cα RMSD < 1.5 Å, pLDDT > 75, and catalytic residue Cα RMSD < 1.0 Å. In the case of final round N+1 oxyanion hole designs, a modified version of Initial Guess AF2 was used to predict designs with sparse template information provided (see Supplementary Methods).

Designs that passed AF2 filters were subsequently analyzed using PLACER. PLACER is a denoising neural network trained on X-ray and EM structures from the PDB to recapitulate the correct atom positions from partially corrupted input structures provided the atom type and bond connectivity is known. PLACER predictions were done for a spatial crop of 600 atoms closest to the active site. The inputs to the network included the protein backbone coordinates within the crop and the amino acid sequence with side chain coordinates randomly initialized around the respective Cα atoms. For proteins without a crystal structure, the AF2 model was used. For every designed protein, we modeled 5 reaction states representing the chemical modifications the catalytic serine undergoes in the course of the reaction: 1) apo, 2) substrate bound, 3) tetrahedral intermediate 1 (TI1), 4) acylenzyme intermediate (AEI), and 5) tetrahedral intermediate 2 (TI2). We used 50 different seeds to generate an ensemble of 50 PLACER models for each reaction state (apo, substrate bound, TI1, AEI, and TI2). For each of the 50 models in a given ensemble, the presence and geometry of key hydrogen bonds in each individual model (see Supplemental Methods) were determined. To analyze native hydrolases with PLACER, a set of native crystal structures was collected (34) (PDB IDs: 1ACB_E, 1C5L_H, 1H2W_A, 1IC6_A, 1IVY_A, 1PFQ_A, 1QNJ_A, 1QTR_A, 1ST2_A, 2H5C_A, 2QAA_A, 3MI4_A, 5JXG_A), the active site locations identified, and the aforementioned process applied.

### In-gel fluorescence screening with activity-based probes

DNA encoding the designed proteins was ordered from IDT as eblocks and the GoldenGate method used to clone them into vector LM627 (addgene), which contains a C-terminal SNAC tag followed by a hexahistidine-tag. Resulting plasmid was transformed into BL21(DE3) cells and grown overnight in 1 mL of LB supplemented with 50 μg/ml kanamycin. For expression, 100 μL of overnight culture was used to inoculate 1 mL of LB media and grown for 1.5 hours at 37°C on a Heidolph shaker and then 10 μL of 100 mM IPTG was added and cultures were shaken at 37°C for an additional 3 hours. Cultures were centrifuged at 4000*g* for 10 minutes and supernatant removed. Cell pellets were resuspended in 200 μL 20 mM HEPES (pH 7.4), containing 50 mM NaCl, 0.1 mg/mL lysozyme, and 0.01 mg/mL DNaseI. After 15 minutes, lysates were frozen in liquid nitrogen and subsequently thawed at room temperature. 10 μL of lysate was incubated with 1 μM FP-TAMRA probe (10 μL of 2 μM stock in lysis buffer) for 1 hour at room temperature before quenching using 2x Laemmli sample buffer. Labeled samples were heated at 95°C for 5 minutes and 10 μL of each sample was separated on a BioRad AnykD Criterion precast gel and in-gel fluorescence was imaged using a LI-COR Odyssey M imager. Gels were subsequently stained with coomassie blue and imaged again.

### Lysate screening

DNA encoding the designed proteins was ordered from IDT as eblocks and cloned by the GoldenGate method into vector pCOOL1 which contains a C-terminal mScarlet-i3 fusion and to enable normalization of activity in lysate. Resulting plasmid was transformed into BL21(DE3) cells and cultures were grown overnight at 1 mL scale in 2 mL deep well 96-well round bottom plates on a Heidolph shaker at 1300 rpm and 37 °C. For expression, 50 μL of the overnight cultures were used to inoculate 1 mL of autoinduction media in 2 mL deep well 96-well round bottom plates and incubated at 1300 rpm and 37 °C for approximately 24 hours. Cultures were centrifuged at 4000*g* for 10 minutes and supernatant decanted, washed with buffer (20 mM HEPES, 50 mM NaCl, pH 7.4), and incubated on a Heidolph shaker at 1300 rpm at room temp for 5 minutes to resuspend. Plates were centrifuged again at 4000*g* for 10 minutes and supernatant decanted. For lysis, cell pellets were resuspended with 500 μL of lysis buffer (20 mM HEPES, 50 mM NaCl, 0.01 mg/mL DNAseI, 0.01 mg/mL lysozyme, 1 mM EDTA, 0.1% triton X-100) and incubated for 2 hours on a Heidolph shaker at 1300 rpm and 37 °C. Plates were centrifuged at 4300*g* for 30 minutes and supernatant collected for screening. For activity screening, 4 or 6 μL of lysate was aliquoted into microtiter plates and reactions initiated by addition of 36 or 54 μL of buffer containing 111.1 μM 4MU-Ac or 4MU-Bu, 20 mM HEPES, 50 mM NaCl, pH 7.4, 5% DMSO. Volume sizes were modified depending on plate type used, where half-area plates were used for 40 μL reaction volume and full-area plates were used with 60 μL reaction volume. Upon addition of substrate, microtiter plates were measured once for mScarlet-i3 signal and then subsequently monitored continuously for the generation of 4MU (ex: 365 nm, em: 445 nm).

### Protein expression and purification

Genes encoding the designed proteins were ordered from IDT as eblocks and cloned via the Golden Gate method into vector LM627 as previously described (66). Resulting plasmid was transformed into BL21(DE3) cells and grown overnight in 1 mL of LB supplemented with 50 μg/ml kanamycin, after which 500 μL of overnight was used to inoculate 50 mL of autoinduction media (67), which was grown 4–6 hours at 37 °C and then overnight at 18 °C. Cultures were spun down at 4000*g* for 15 minutes, and supernatant decanted. Cell pellets were resuspended in 25 mL of cold wash buffer (40 mM imidazole, 500 mM NaCl, 50 mM sodium phosphate, pH 7.4) with 1 mg/mL lysozyme and 0.1 mg/mL DNAse I. Cell slurries were sonicated on ice for 2.5 minutes at 80% amplitude, 10s on 10s off. The resulting lysate was centrifuged at 14000*g* for 30 minutes and the supernatant was applied to 1 mL of Ni-NTA resin equilibrated with wash buffer. The resin was subsequently washed with 15 mL of wash buffer 3 times and once with 400 μL of elution buffer (400 mM imidazole, 500 mM NaCl, 50 mM sodium phosphate, pH 7.4) followed by elution with 1.3 mL elution buffer. The eluate was purified by size-exclusion chromatography on a Superdex 75 Increase 10/300 GL with running buffer of 20 mM HEPES, 50 mM NaCl, pH 7.4. Samples were either used immediately in downstream experiments or snap frozen in liquid nitrogen and stored at −80 C. Protein molecular weight was confirmed by LC-MS.

### Kinetic analysis

To characterize hits identified from in-gel fluorescence and lysate screens for catalytic turnover, we incubated purified protein samples with fluorogenic substrates 4MU-Ac, 4MU-Bu and 4MU-PhAc. Kinetic screens were either performed in 40 μL reaction volumes in 96-well half area plates or 60 μL reaction volume in 96-well full-area plates. Protein and substrate were prepared in 20 mM HEPES, 50 mM NaCl, pH 7.4, 5% DMSO. Either 4 or 6 μL of enzyme was added to microtiter plates and the reactions were initiated by addition of substrate (36 or 54 μL). Generation of the fluorogenic product 4MU was monitored continuously (excitation 365 nm, emission 445 nm). Analysis of the resulting data were carried out using custom scripts (see computational methods). In cases where single-turnover activity was observed, initial velocities were used to determine *k*_2_/*K*_m_. For those designs that displayed a clear burst phase followed by a slower steady-state rate, straight-line fits of the steady-state velocities were used to determine Michaelis-Menten catalytic parameters.

To determine the uncatalyzed reaction rate in assay buffer (20 mM HEPES, 50 mM NaCl, pH 7.4, 5% DMSO), substrate was diluted in buffer alone and rates determined at multiple substrate concentrations, after which the rate was determined from fitting [S] versus rate with an equation of the form rate = *k*_buffer_[S].

### Crystallography

Proteins for crystallography were prepared as described above, but SEC was done with SNAC tag cleavage buffer (68). After SEC, protein eluate was incubated with 500 mM guanidinium hydrochloride and 2 mM NiCl_2_ overnight at room temperature to remove the C-terminal His tag. The SNAC cleavage reaction was applied to a nickel column equilibrated with wash buffer to remove any uncleaved product and resulting eluate applied to a Superdex 75 Increase 10/300 GL column with 20 mM HEPES, 50 mM NaCl, pH 7.4 as the running buffer. Samples were concentrated and stored at −80° C or immediately used for crystallization. Crystallization screening was performed using a Mosquito LCP by STP Labtech and resulting crystals were harvested directly from the screening plate. Crystallization conditions for each design were as follows: **r0-n8** (15 mg/mL) in 0.1 M Bis-Tris pH 5.5, 25% (w/v) PEG 3350, **r3-super** (50 mg/mL) in 0.2 M Potassium fluoride, 20% (w/v) PEG 3350, **r3-win** (42 mg/mL) in 0.1 M Sodium acetate pH 4.6, 8% (w/v) PEG 4000, **r4-win1** (54 mg/mL) in 60% v/v Tacsimate pH 7.0, **r4-win31** (60 mg/mL) in 0.2 M di-Ammonium tartrate and 20% (w/v) PEG 3350, and **r4-dadt1** (27 mg/mL) in 0.1M Potassium chloride, 0.02 M Tris pH 7.0, and 20% PEG4000. Data were processed with XDS (69), phased and refined with Phenix (70), and model building performed with COOT (71). Percent Ramachandran favored, allowed, and outliers for each structure are as follows: **r0-n8** (98.21, 1.79, 0.00), **r3-super** (99.37, 0.63, 0.00), **r3-win** (97.99, 2.01, 0.00), **r4-win1** (99.68, 0.32, 0.00), **r4-win31** (99.36, 0.64, 0.00), and **r4-dadt1** (100, 0, 0). Coordinates are deposited in the PDB with PDB IDs of 9DED (**r0-n8**), 9DEE (**r3-super**), 9DEF (**r3-win**), 9DEG (**r4-win1**), 9DEH (**r4-win31**), and 9MRB (**r4-dad_t1**).

### Mass spectrometry

Intact mass spectra of protein samples were obtained by reverse-phase LC/MS on an Agilent G6230B TOF after desalting using an AdvanceBio RP-Desalting column. Deconvolution using a total entropy algorithm was performed using Bioconfirm. In some cases, protein samples (1 mg/mL) were incubated overnight with substrate (300 μM) in SEC running buffer at room temperature prior to mass spectrometry analysis.

### Structural similarity search of the PDB and AFDB

To assess the structural novelty of our designed enzymes, we used FoldSeek (72) to compare our crystal structures and select design models against all available databases. Searches were performed in TM-align mode and the highest TM-score hit was used for structural comparison.

## Supplementary Material

supplementary material

## Data Availability

Crystal structures are available in the protein data bank with PDB IDs 9DED (**r0-n8**), 9DEE (**r3-super**), 9DEF (**r3-win**), 9DEG (**r4-win1**), 9DEH (**r4-win31**), and 9MRB (**r4-dad_t1**). Code and example execution commands for each step of the design pipeline is available for download in the provided repository and will be made publicly available at https://github.com/laukoag/serine-hydrolase-design and in Zenodo (73).
