# Supplementary material for: Computational design of serine hydrolases
Source: Science. Author manuscript; Available in PMC 2025 Jul 24. (PMC12288761; doi:10.1126/science.adu2454)
Supplement: Supplementary file 1 [file NIHMS2090701-supplement-supplementary_material.pdf]

Science
AAAS

# Supplementary Materials for

## Computational design of serine hydrolases

**Authors:** Anna Lauko, Samuel J. Pellock, Kiera H. Sumida, Ivan Anishchenko, David Juergens, Woody Ahern, Jihun Jeung, Alex Shida, Andrew Hunt, Indrek Kalvet, Christoffer Norn, Ian R. Humphreys, Cooper Jamieson, Rohith Krishna, Yakov Kipnis, Alex Kang, Evans Brackenbrough, Asim K. Bera, Banumathi Sankaran, K. N. Houk, David Baker*

*Corresponding author: spellock@uw.edu, dabaker@uw.edu

**The PDF file includes:**

Supplementary Text
Figs. S1 to S20
Tables S1 to S6
References 74-76

**Supplementary Text**

Motif generation via conformational sampling

To generate constellations of histidine rotamers around the serine backbone motif, distances, angles, and torsions were first specified in enzyme constraint file format along with sampling ranges and frequencies for the serine-substrate and serine-histidine interactions. These were input to a script which (1) sampled probable histidine rotamers from the Dunbrack library at the specified geometries with respect to the serine nucleophile and substrate, (2) built out protein stubs in helical or strand conformation flanking the histidine using Rosetta, (3) and filtered for clashes between the substrate, serine stub, and histidine stub before outputting these conformations as PDB files. The code and a detailed description of this script can be found here: https://github.com/ikalvet/invrotzyme .

Evaluation of hydrogen bonds in PLACER predictions

Hydrogen-bonding interactions in PLACER predictions were evaluated by the measuring of distances, angles, and torsions between the acceptor and donor heavy atoms. Upper and lower bound cutoffs for each parameter (distance, angle, dihedral) were used to determine if the interaction constituted a hydrogen bond, depending on the hybridization of the participating heavy atoms.

Active site composition and geometric features

To identify potential strategies for improving $k_{cat}$ in our designed hydrolases, we performed a comparative analysis of the active sites of our designs and natural hydrolases, identifying a number of deviating features in the catalytic triad, oxyanion hole, and surrounding molecular environment. Differences in active site makeup could contribute to decreased $k_{cat}$ values in our designs. In nature, the catalytic aspartate or glutamate is supported by a network of additional hydrogen bonds from surrounding sidechains and backbone amides *(61)*, a feature rarely seen in our designs, which have at most one sidechain hydrogen bond to the catalytic aspartate. This highly polar environment helps aspartate remain in a charged state to stabilize the catalytic histidine, and clearly plays an important role in our designs as well, as evidenced by the significant effect of knockout mutants of these second shell contacts on activity (fig. S12). Although some notable exceptions exist, such as the serine protease subtilisin, in most natural serine hydrolases, at least 7 two backbone amide groups comprise the oxyanion hole, while all but one of the active designs herein contain one backbone amide and one sidechain hydrogen bond. Sidechains are expected to be significantly more challenging to preorganize than backbone amides due to additional rotatable bonds, and crystal structures of our designs often show deviations from the designed oxyanion hole sidechain conformations. Indeed, the design with the highest $k_{cat}$, **momi120**, has two backbone amide hydrogen bonds. Geometric deviations within the catalytic triad and oxyanion hole could also account for the differences in $k_{cat}$ between our designs and native serine hydrolases. In nature, the catalytic aspartate and histidine form a particularly ideal hydrogen bond, characterized by short distances and near linear bond angles *(33)*, while crystal structures of our designs display longer distances and deviations from ideal angles. Alignment of the active sites of natural enzymes and our active designs also reveals a difference in the angle of approach of the histidine to the serine in our designs (fig. S16). This shift may affect the role of the histidine in shuffling protons between the serine, substrate and

hydrolytic water. Finally, the oxyanion hole hydrogen bonds of natural hydrolases are positioned to make out-of-plane interactions with the ester carbonyl of the substrate, which has been hypothesized to lead to selective stabilization of the oxyanion containing transition states and intermediates over the ground state *(34, 51, 52)*. Our designs exhibit hydrogen bonds that are closer to in-plane hydrogen bonds that may over-stabilize the sp2 ground state of the substrate carbonyl.

Design of catalytic triad containing proteins in NTF2 scaffolds

Theozymes containing catalytic triads and one or two sidechain oxyanion hole residues were docked into a set of hallucinated NTF2 scaffolds *(40)* using RosettaMatch, designed using three cycles of RosettaDesign with enzyme constraints applied. Designs were input to AF2 for single-sequence structure prediction as described in the Methods ("Filtering"), and filtered for a global Cα RMSD < 0.7 Å, pLDDT > 93, and catalytic residue Cα RMSD < 1.0 Å.

## Structure generation with a new variant of all-atom RFdiffusion

In this study, we follow the familiar protocol of generating protein backbone structures with a diffusion model, followed by a fixed backbone sequence design step. The diffusion model we use is a newly trained variant of RFdiffusionAA, which we call 'CA RFdiffusion' (as in, alpha-carbon). In this section, we detail the training and inference protocols for this model. All training and inference code will be released upon manuscript publication.

Introduction

CA RFdiffusion comprises a set of two models, a diffusion model and a "refinement" model, that are both fine-tuned versions of the same pretrained RosettaFold All-Atom structure prediction network. There are two main differences between CA RFdiffusion and any previous variant of RFdiffusion(AA). First, the diffusion model itself produces only alpha-carbon coordinate positions, instead of full N-CA-C frames with both a translation and orientation (as in RFdiffusion *(36)* and RFdiffusionAA *(41)*). This means that a second step after generating the "CA trace" must be taken to obtain a fully constrained polypeptide backbone which can be assigned a sequence (one could also design sequences from CA coordinates alone, but we did not attempt this). We perform a second "refinement" step by training a second separate model to produce a full protein backbone given just the trace of alpha carbons.

The second main difference is that instead of taking in motif structure information via static 3D coordinates supplied to the network (as is the case in RFdiffusion and RFdiffusionAA), CA RFdiffusion takes in motif structure information via the available inter-residue pairwise distance and orientation input. In RosettaFold All-Atom (and other previous structure prediction networks) this input is normally used for supplying structural information of proteins homologous to the query sequence during structure prediction (see *(41, 74, 75)*). For CA RFdiffusion, we use this input to encode motif structures as their pairwise distances and orientations, and have the network reconstruct the motif in its output 3D predictions. It is noteworthy that at least one other group *(76)* has developed an analogous motif-scaffolding technique concurrently and independently of the method we describe here. One main benefit of this motif input style is that, as Lin et al. *(76)* also point out, a user is able to present multiple

discontiguous motifs to the network for scaffolding without specifying their relative rigid body transform (as is mandatory, by definition, for methods that take in 3D coordinates of the motif).

Training CA RFdiffusion

Here we detail the two-part training of CA RFdiffusion, which comprises training a diffusion model for generating CA traces, followed by training a "refinement" model which produces full protein backbones from the CA traces.

Architecture

Training the diffusion model for CA RFdiffusion is similar to *(36)*, but there are several differences in the inputs to the network and loss function. We begin with the architecture and pretrained weights of RoseTTAFold All-Atom *(41)*. To this architecture, we expand the dimensions of the template inputs (see Krishna et al. 2024 for architecture details) to include one additional per-token and per-token-pair indicator feature. These denote whether or not a token, or pair of tokens, is part of a motif being scaffolded by the network. During training, we supply three distinct templates (analogous to three separate homologous structure inputs during structure prediction), as shown in Table S3.

We initialize the weights associated with the expanded feature dimensions as all zeros, such that predictions with the expanded architecture completely ignore the new features at first. However, as training progresses, the network can learn to correlate the motif indicator features with the perfect motif information supplied in template #3, helping distinguish that particular structural input from the rest of the structural inputs.

Preparing training examples

Training examples are prepared in two stages: First, a dataset is selected to draw a structure from; either (A) PDB protein monomers without small molecules, or (B) PDB protein monomers in complex with small molecules. Then, a masking strategy is chosen for the drawn structure. The masking strategies available to either dataset are not the same, and they are outlined in Table S4 below. Once the dataset and masking strategy are chosen, any structural components which are considered motif (any ligand, and unmasked protein fragments) are encoded in the motif (third) template with appropriate indicator features.

To noise a selected example, we uniformly sample from the discrete set of times $t \in [1,200]$ and then adopt precisely the CA-coordinate noising strategy employed by *(36)* to noise the CA positions of each N-CA-C frame in the backbone and all atoms in any ligand. We do not use any noising strategy for the frame orientations, and instead set all residue frame orientations to the identity rotation, which removes any information about the native structure contained in the frame orientations. It is noteworthy that this step breaks the equivariance of a network forward pass, because the process of setting all frame orientations to an arbitrary orientation is not equivariant to arbitrary rotations of the input molecules. A summary of training example generation hyperparameters can be found in Table S5. It is worth emphasizing that because the motif information is encoded in the template inputs to the network (described above), all residues and small molecule atoms are noised in 3D space, and the network is tasked with reconstructing the motif in its output--a well-defined problem since the motif is templated.

Loss function

The loss function to train the diffusion model for CA RFdiffusion is:

$$L_{diffusion} = W_{disp}L_{disp} + W_{disto,anglo}L_{dist,ang} + W_{FAPE,prot}L_{FAPE,prot} + W_{FAPE,prot-sm}L_{FAPE,prot-sm} + W_{FAPE,sm}L_{FAPE,sm}$$

Where $L_{disp}$ is the CA coordinate displacement loss (as described in *(36)*), $L_{dist,ang}$ is the pairwise inter-residue distogram and anglogram cross entropy loss (as described in *(36)*), $L_{FAPE,prot}$ is intra-protein frame-aligned point error (FAPE) *(50)* loss on the motif--applied only to pairs of residues which were supposed to be constrained with respect to each other in the motif template definition using Algorithms 1, 2, and 3. $L_{FAPE,prot-sm}$ is the inter-protein-ligand FAPE loss, only computed for frame-point pairs between motif amino acids and ligand atoms (recall, ligand atoms are always considered motif). $L_{FAPE,sm}$ is the FAPE associated with ligands internally only. The values of their respective weights can be found in Table S5.

While $L_{disp}$ and $L_{disto,anglo}$ are identical to how they were defined and applied in *(36)*, the application of FAPE losses on protein and ligand motif fragments is new to the CA RFdiffusion diffusion model. The FAPE losses were applied to encourage precise and robust reconstruction of ideal motif geometries in the final output of the network. Note that because FAPE by definition scores the quality of an N-CA-C rigid frame orientation, the diffusion model will produce full backbone heavy atom predictions for motif regions, and CA-only predictions in non-motif regions. All small molecules are considered to be a motif and thus encoded in the template structure input.

Training the refinement model

Because the diffusion model only produces CA positions in non-motif regions, a second model ("the refinement model") was trained to take in CA-only descriptions of backbones and produce a refined backbone with all N-CA-C positions and orientations fully described. In short, a model was trained to take in natural protein structures with slightly noised CA positions and random N-CA-C frame orientations, and reconstruct the native backbone heavy atoms. Note this is not a diffusion process, but instead a single shot structure noising and refinement.

Architecture

The architecture of this model is precisely identical to that of the diffusion model described above.

Preparing training examples

Training dataset, masking strategy and motif templating are very similar to that described above for the diffusion model. The only differences are (1) Instead of noising the resulting structures in a forward diffusion process, a single instance of 3D Gaussian noise is added to all CA atoms and ligand atoms in the structure, and the orientation of N-CA-C frames is randomized. (2) The model is trained in two stages in which the datasets sampled are different: We trained for the first 4 epochs with variance 1.0 Å$^2$ on the PDB monomer only dataset (i.e., no ligands), then for 3 additional epochs with variance 1.5 Å$^2$ on both the PDB monomer only dataset and the monomer + ligands dataset. For N-CA-C frame orientation randomization, we simply use the scipy.spatial.transform.Rotation object to produce random rotation matrices for our frames

via Rotation.random(L), and orient them accordingly. These examples are then fed to the model, and it is tasked with reproducing the native protein structure.

Loss function

The loss function for refinement is very similar to $L_{diffusion}$ defined above:

$$L_{refinement} = L_{diffusion} + W_{FAPE,prot(non-motif)} L_{FAPE,prot(non-motif)} + W_{pLDDT} L_{pLDDT}$$

Where $L_{FAPE,prot(non-motif)}$ is the FAPE loss associated with any non-motif regions (which are always protein only regions), $W_{FAPE,prot(non-motif)}$ is its corresponding weight, $L_{pLDDT}$ is the loss associated with predicting the LDDT of the refined structure (see *(41)* for details of function) with weight factor $W_{pLDDT}$ Additionally, for refinement training we set $W_{disp} = 0$ (i.e., no loss from the coordinate displacement loss function) and let FAPE serve as the dominant loss signal. See Table S5 for the weight values for specific components of the loss function.

Running inference

Inference is run by running a denoising diffusion trajectory with the trained diffusion model, templating a desired motif and constraining inter-motif-chunk DOFs as the user desires (in this study, we constrain all components of the motif with respect to all others). The output from this denoising trajectory is then fed into the refinement model, which receives a template of the original perfect motif, as opposed to a template of the motif as it was reconstructed by the diffusion model. The refinement model then produces a full heavy atom prediction of the backbone, usually reconstructing the motif to within less than 0.2 Å of the native/input. For sidechains within the motif, we rebuild the rotamers from the input motif onto the corresponding residues in the output, using the sidechain dihedral angles from the original motif, and ideal bond lengths and angles. At this stage, the output is ready for sequence design.

Sequences of key designs

**>n8**
EEERFRAYYERYFAALAARDYETLLEILREFGVAKLVLNGREFASPEEAVQWARDTGLR
FLRLIRERYEDGVYTVEDIVSRDDGRYYRHTSTLRRQPDGSYVQYSQLELL
**>shh_25**
LSEEEVEKAIKEIKEKFEKLAKEIEKLVAEGADRDTLVERLVKYAASLGAASASPETSTPE
QYAAVREIFRALADAILDGRWEEAGERLVEATVRHTQALIDAARAAGRDELVPALRRL
GLALAEAIFDILREYIAKKTGDAAAAERYKETYLARLRAAL
**>rem6507**
MSEEEVERRIREIHAMFEKLEEKIDELVAAGADVETLTSELVRFAASLGADSANPEKSTP
EQYEAVKEILRALAEAIINGKWEEAGKTLVDATVRHTGALIERARAAGREDEVPALRRL
GEALLRSTLEILRKYIEKKTGDKELADKYFETYLAEFRKKL
**>win**
MTEEELDQAVEDFLRVHSELVHRLAGDPPDELFQRLDRFVTDAIIEGNPERRDEIKADLA
RAARVFGEALERDITTPEDFNAFLRELGPEAVELVSTFTQQFVDVIRGDPQAVAEHLNIS
LEDVARLAEAGEAAIERGEGASLGVHRELRRIEARRNS
**>super**

SENEKLVEKVLEATRRIAREEAVKYKDAFLRAYRARDGAGLRRVITGLFSKVDSRLYKE
VLTDVPTIVALQRRAGVDITPEQAQEILDNYDNEKHTAAVMDETFALLARAAATQASYE
ELLAAAPSGSVILALEVLRVLLEINNLSWREVLPLLALAAAS

**>win1**

LSDRELEASLQAFFEVHTRLVHRLAGIEPDPRFEILDKYIFRQIVADNPEEREKIRLDYGR
AAEIFRDALARDITTPEAFNAYLEALGPDAVRTVQDLTRRFVDVIRADPEAIAKLLNISKE
DVQGLARAGEAAIERGEGASLGVLRELRKIEKKRNE

**>win11**

MTQAELDDGVTRFIQVHNELVHRLAGVEPDERFIKLDVYVTNEIVNSDPTKAAERKQAL
ARAADVFSEALARDIRTAEEFNAFLEELGPEAVELVADLTRAWVEVIESDPEKIAQLLNI
SVEEVEELAEAGRRAIEEGRGASIGVLLKLREIEKIRSS

**>win31**

MTEEELEKGVEDFLVVHGKFVHRLAGIPPNAKFQALDKYITNQIVESDPSKEKEIKKAFG
DAAKILRDALARNITTPEEAQAFLRDLGPWAVDLINTITRRYVDVIEKNPEGVAEILGISL
EEVRELAEAGRRAIEEGEGASLGILRKILELEAERAK

**>dadt1**

MTEEEKAQAVEDFHKYHTALVVRLAGVPPHERYERLDRWITEQLIYGDESKRDEWIDA
LAEAAGIFKEALERNITTPEEFNAFLKEKGKRAVDLVATLTNAYIAVLEGDPEAIARFLGI
SLEEVQEIIDAARKAVKEGRGASIGIYTKLRELEERRAA

**>charliet2**

AAEAKARWKKAVEMFKKLSDKLLEAGAGVSPAFAVSIAYAAGIITEEQVYSTIEGVIAK
VKADPERWQKEVAELFTTYKDDLDAFVEKHTELVKRLLGDSLPDEVFELFKEKDKEVL
EKIPKELWEKVTSLFEEGNYEEANKLIQKAYREYALEILEGEYNSL

**>kent1**

AEEEAVRAVEERDDEALGRALFGAADAGDLDGATRAHTAAVLKGRELGIDAGSLAIYD
VVAEYVRTGKKPPKEEAEKLVLGYAKRDVERRADGTPLERVLAEATLVFAKTFLDNYD
EIFAELEKDPSLTPDEAFAVADRFLWDVMAARGEWDKLLAAYRAAVG

>**win1_b1**

MTEAERQELLDRAVSAHQRFVFDRLGVERDPRYEALDRFTVDIIVGENKERRDEVYRDL
ALLREGIELFYKQNIPMKEITKEDIEKLPKEMYDAFTRLTDYFIEVVEERKEEIAKLLGFT
LEEIDRIVEAGKKAIEEGQGASIGVATAIREIAAEREA

**>momi**

AEEEAVRAVEERDDEALGRALFGAADAGDLDGATRAHTAAVLKGRELGIDAGSLAIYD
VVAEYVRTGKKPPKEEAEKLVLGYAKRDVERRADGTPLERVLAEATLVFAKTFLDNYD
EIFAELEKDPSLTPDEAFAVADRFLWDVMvAARGEWDKLLAAYRAAVG

**>superfast**

SEEEELINKIEEETKKLAKEVAEKYKDAFKAAFDAEDGKGLRDVLTEAWREVDIKIWKK
FFLNPDDPEGGKRNYALVTKVNRERAKELGIEEVAERVEKAISSAKNLEEIVDNHTEIVL
ELSFAQLVEAAKEKADFDWIYENAPSISSRLFFIVLKELVEINNLSWVDVLPLLARAATA

**>supercool**

EKDKELHKKVFDFVDKVAREVVAGYVDALRAAVAARDGAGVRAVLTEAFREVDREL
HKFLTSEENKELFDYVIEYNRRIYLEQNPELSELPEKYKELEKVIRDPDIDKYLEVHVPLV
MEQAFGLLAEAARRRWDWDEVYAAAPSFSVRAFLRVLRVLLEQSDLDWVSTLPLIAEA
ATA

>**momi120**

MIKEYEFPAKKAKTVEEAEKNVEEEIELMKSSGVSFSARRERVVVVGYSLGVFAGIIMF
AANTTYEEALKLAKEIFKEALKNPELIARHLALHRNAETTGKEEWRQDIETWIKIIEKRL
GKPVDKSRIFIATTKEEAVRLAEEAVKLGRAVIYTPPHLLPVAGDEIVEVLTKAGVTVLV
KGGIGSGVPLKVYEA

>**momi120-103**

PVRRHRFPARKANNFEEAVANVERLIEEIRAAGVDFSARKERAVVVGYSLGVVTGMIM
FATGTDFIEALRKALEIGKKVVEEDPEFMERHRKIVTDGNRAEIREDIDYWIEVIEKETGH
PVDRSRIFIAETVEEAVELARRAVELGHAIIVLPPYLIGEAGEAVVEVLTAAGVDVLLMG
GLGSGYPVTIYQA

>**momi120-74**

EVKVTTFPARKATTDEEAKANVEQLIEDIEKSGVTFSADVEKFVVIGYSLGIVTGMLMM
YKKTNFVEALREAMEIFDEIRADPANAPFLERHRRIHENGTKEEILEDIKFWIDHIEKKYG
IPFDRSRIFIALTKEEAVELAKKAVEYGRAIIYLPPSLIEEAGDEVVEVLANAGVQVLMM
GGLGSGYPVRIYEA

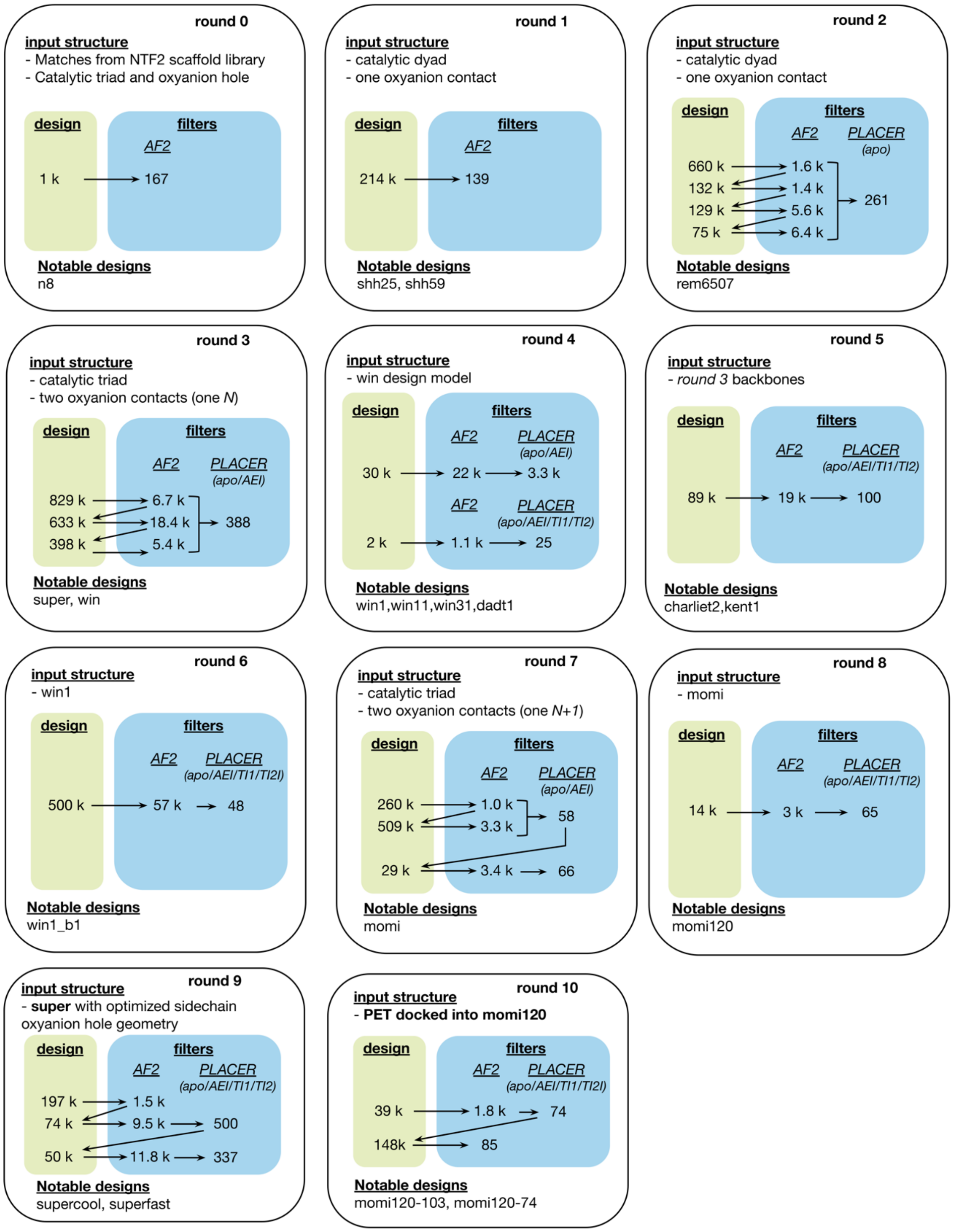


**Figure S1. Summary of design rounds.** Numbers represent generated designs (green boxes) or number of designs passing filters (blue boxes). Diagonal arrows indicate designs that passed filters in a previous round and were redesigned again with LigandMPNN and FastRelax to generate more designs. Key designs described in the main text are listed in the notable designs section.

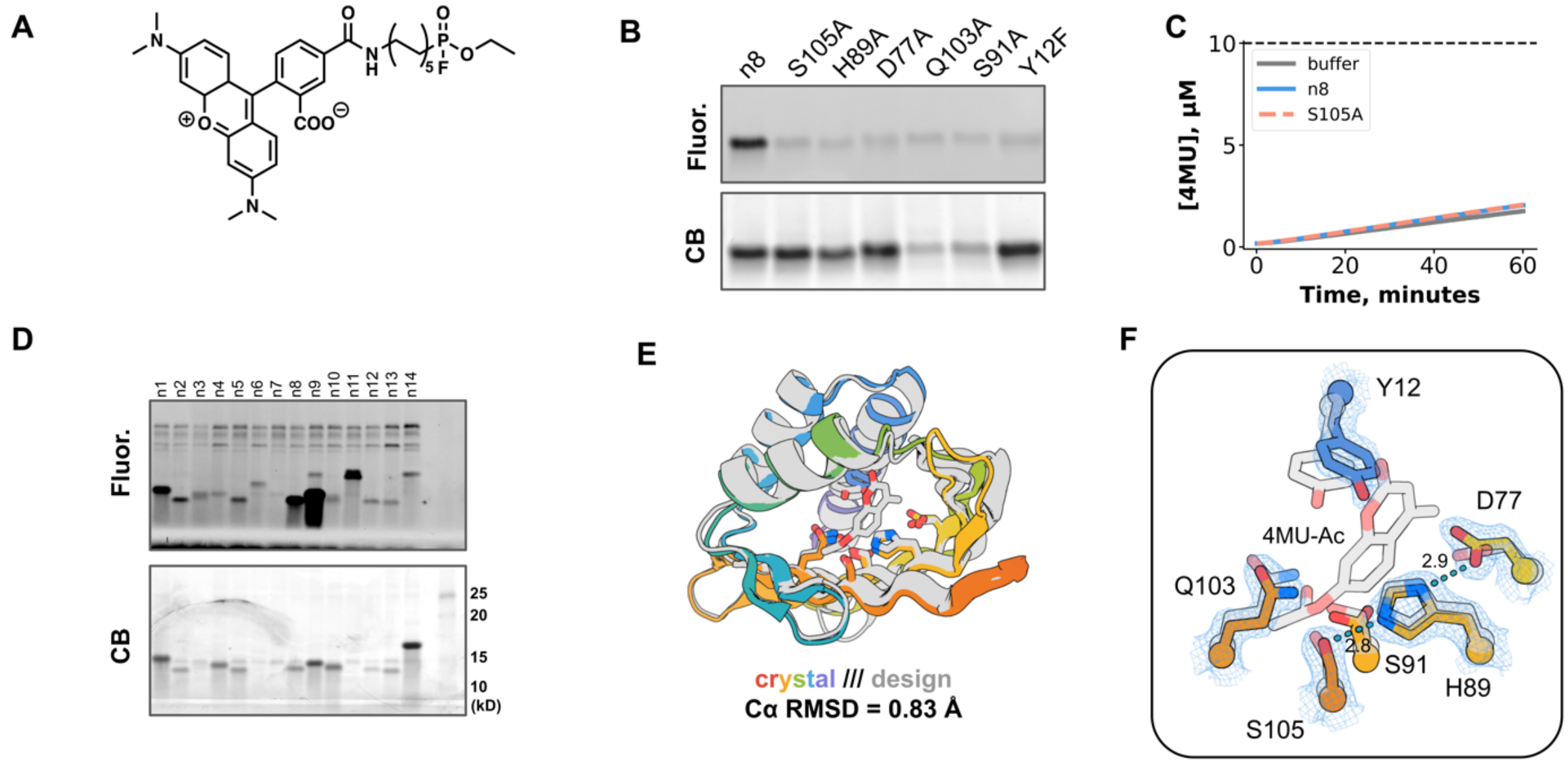


**Figure S2. Serine hydrolase design and characterization with hallucinated NTF2s.** (A) Chemical structure of TAMRA-conjugated fluorophosphonate probe (FP-probe). (B) In-gel fluorescence of catalytic residue alanine knockouts in probe-reactive design **n8**. Fluor. is fluorescence imaging and CB is Coomassie blue stained. (C) Reaction progress curves of 10 μM **n8** incubated with 100 μM 4MU-Ac in 20 mM HEPES, 50 mM NaCl, pH 7.4. Shaded area represents standard deviation of three technical replicates and dashed line represents enzyme concentration. (D) In-gel fluorescence of NTF2-based serine hydrolase design cell lysates after 1 hour incubation with 1 μM FP-TAMRA. Fluor. is fluorescence imaging and CB is Coomassie blue stained. Molecular weights of designs range from 15 to 18 kD. (E,F) Structural superposition of **n8** crystal structure and design model with 4MU-Ac docked in place of fluorophosphonate probe.

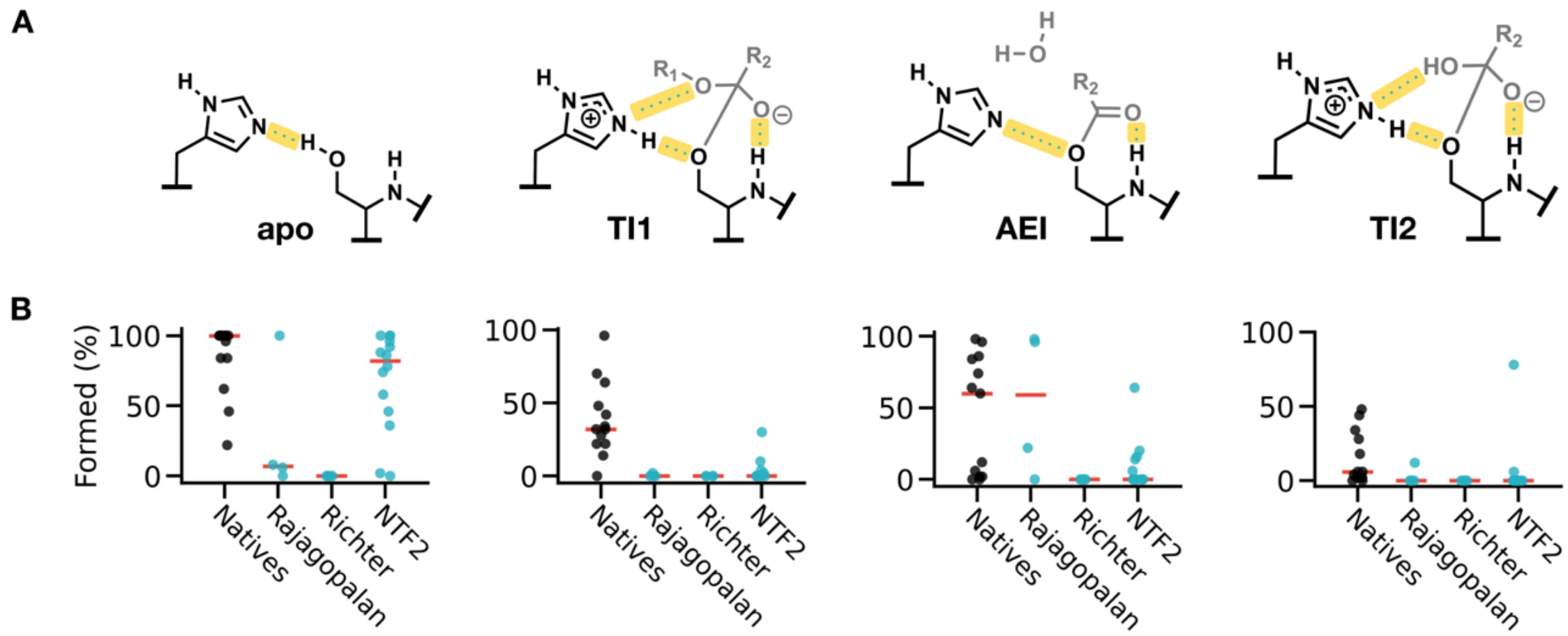


**Fig. S3. Comparing preorganization of native and designed serine hydrolases with PLACER.** (A) Reaction states modeled with PLACER. Key hydrogen bonding interactions are highlighted in yellow. (B) Percent PLACER ensemble frames in which all of the key hydrogen bonding interactions are simultaneously formed for each step (apo, TI1, AEI, TI2 from left to right), with designs in teal, natural hydrolases in black, and medians for each distribution indicated in red. P-values for comparison of natural hydrolases and design distributions were obtained by K-S test and are 0.09, 4.9e-7, 0.03, and 0.0001 for the apo, TI1, AEI, and TI2 states, respectively. Designs labeled "Richter" and "Rajagopalan" were reported in references 6 and 7, respectively. Natural hydrolases (n=13) were obtained from the PDB (see Methods, "Filtering"). Richter (n=4), Rajagopalan (n=4), and NTF2 (n=14) designs were all generated by matching followed by Rosetta sequence design, with the primary difference being the scaffold libraries (native proteins for Richter and Rajagopalan, and hallucinated NTF2s for the NTF2 set) and the input motifs (Cys-His dyads for the Richter set, organophosphate-targeted catalytic triads for the Rajagopalan set, and catalytic dyads and triads targeted toward organophosphates and esters for the NTF2 set) utilized at the matching stage.

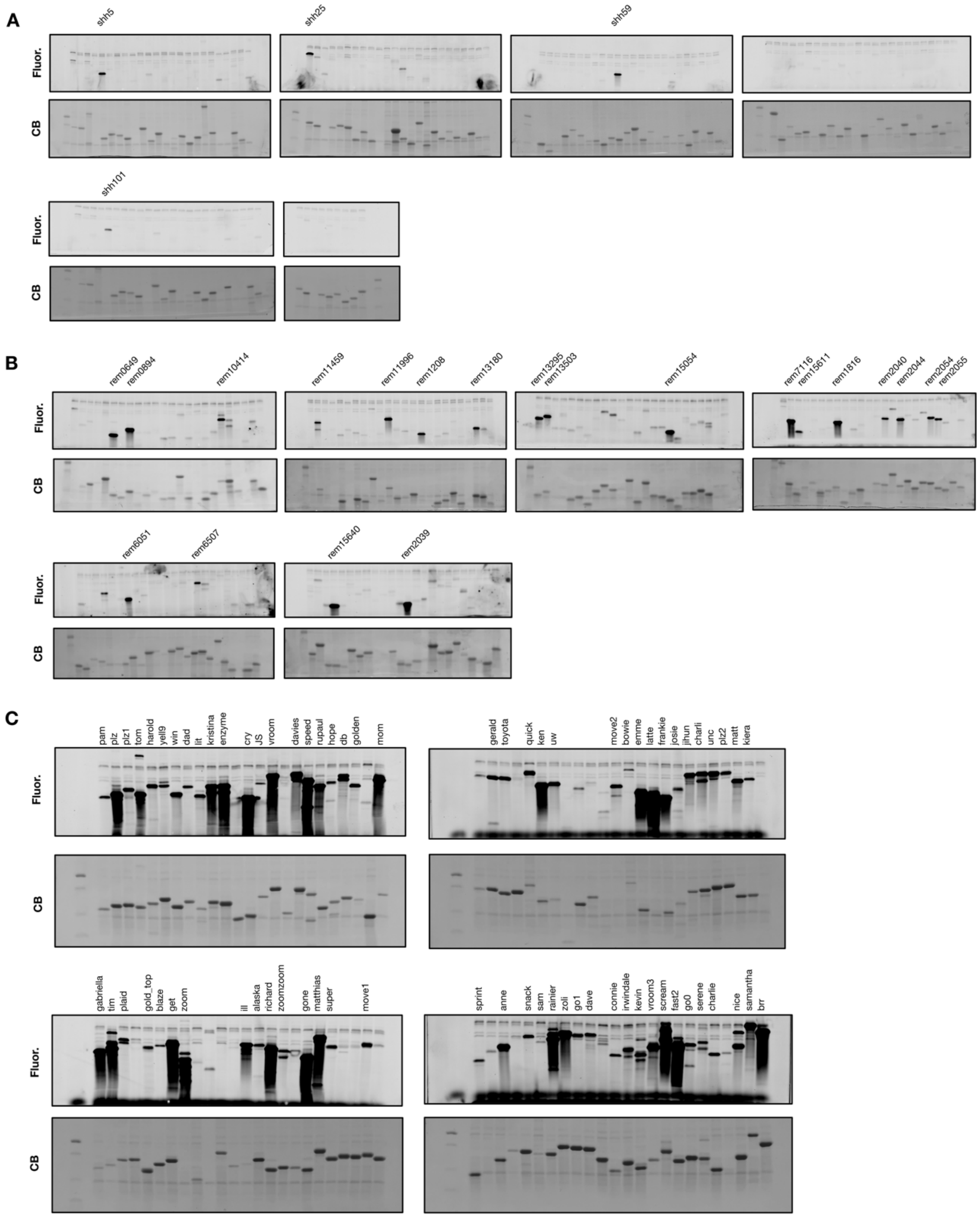


**Fig. S4. In-gel fluorescence imaging with fluorescently labeled fluorophosphonate activity-based probe.** In-gel fluorescence of (**A**) round 1, (**B**) round 2, and (**C**) round 3 designs after 1 hour incubation of cell lysate with 1 µM FP-TAMRA. Fluor. stands for fluorescence and CB for Coomassie blue. Lanes with labels indicate designs that were purified and tested for esterase activity.

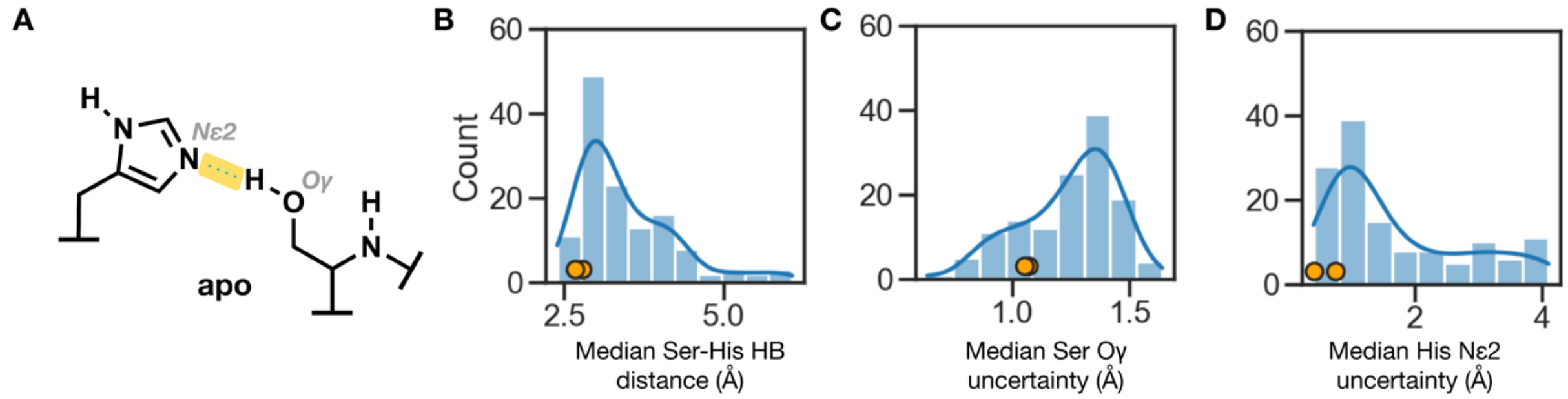


**Fig. S5. PLACER analysis suggests single-turnover designs are more preorganized.** (**A**) Schematic depicting catalytic serine-histidine hydrogen bond interaction. (**B**) Median Ser-His H-bond distance, **(C**) median serine Oγ uncertainty, and (**D**) median histidine Nε2 uncertainty calculated from PLACER ensembles of the apo state for 130 round 1 designs. Yellow points represent the two single-turnover designs, **shh25** and **shh59.**

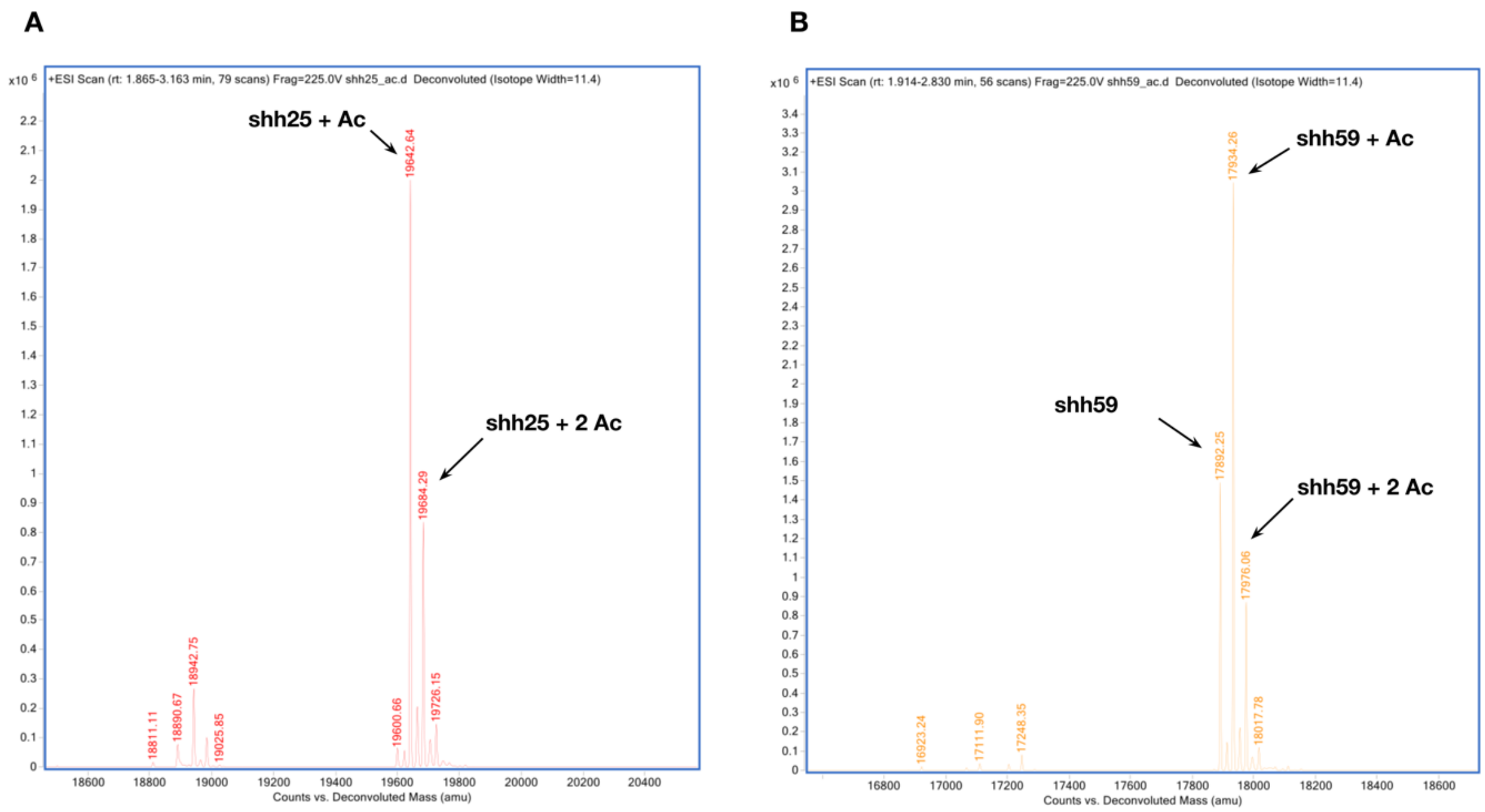


**Fig. S6. Mass spectra of single-turnover designs incubated with cognate ester substrates**. Mass spectra of shh25 (**A**) and shh59 (**B**) after overnight incubation with 100 µM of 4MU-acetate in 20 mM HEPES, 50 mM NaCl, pH 7.4, 5% DMSO buffer.

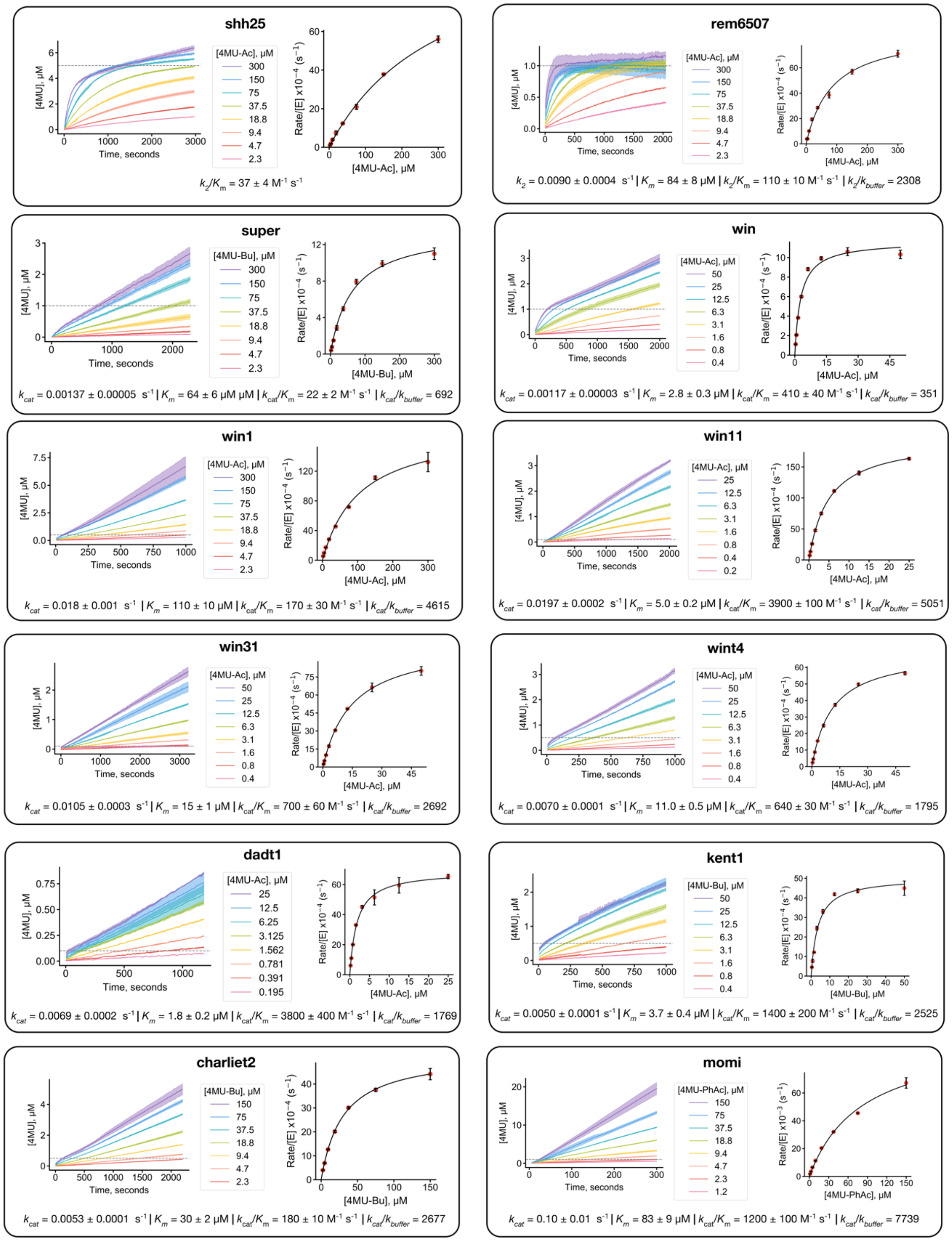


**Fig. S7. Michaelis-Menten kinetics of designed serine hydrolases**. Reactions were performed at 30°C in 20 mM HEPES, 50 mM NaCl, pH 7.4, 5% DMSO assay buffer. Shaded area in progress curves and error bars in Michaelis-Menten plots represent standard deviation of three technical replicates. Horizontal dashed lines represent enzyme concentration.

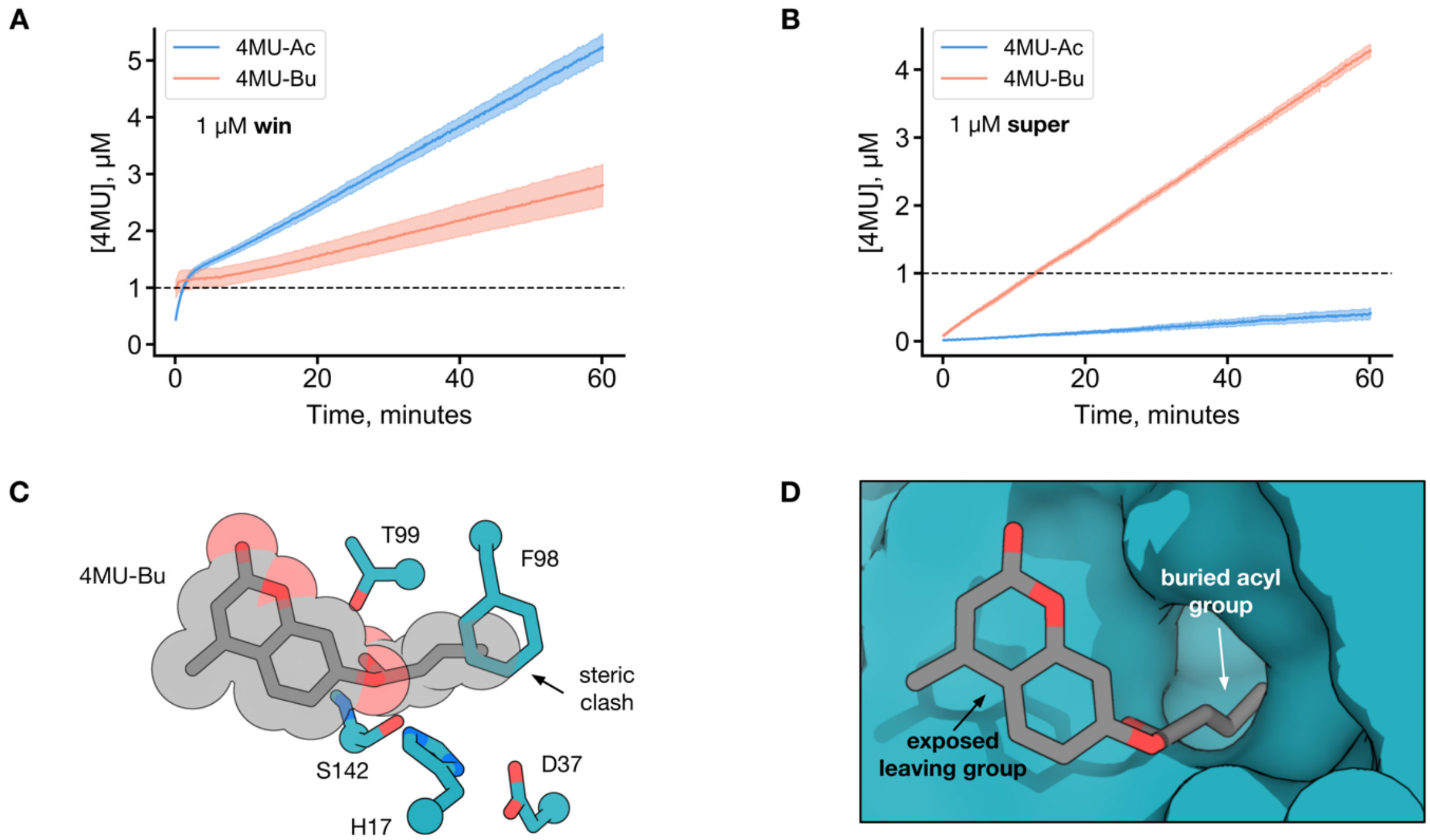


**Figure S8. Substrate selectivity of win and super.** Progress curves of (**A**) **win** and (**B**) **super** incubated with 50 µM of either 4MU-Ac or 4MU-Bu in 20 mM HEPES, 50 mM NaCl, pH 7.4 at 30°C. Dashed line indicates enzyme concentration and shaded area represents the standard deviation of three technical replicates. (**C**) **win** crystal structure with 4MU-Bu modeled in the active site. (**D**) Surface representation of **super** crystal structure with 4MU-Bu docked into the active site.

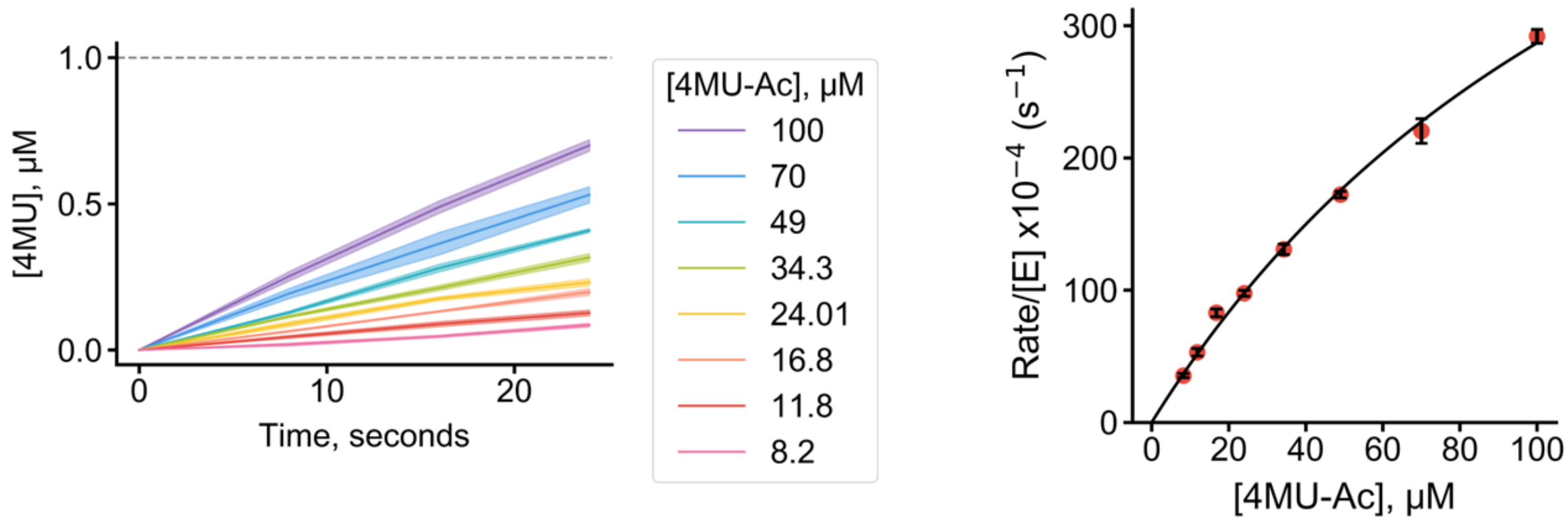


**Figure S9. Burst phase kinetics of win.** Progress curves (left) and Michaelis-Menten plot (right) of the burst phase of **win** (1 μM) incubated with up to 100 μM of 4MU-Ac does not reach a saturating velocity, suggesting $K_s >> K_m$ ($K_m$ = 2.8 μM). Dashed line represents enzyme concentration and shaded area represents standard deviation of three technical replicates in the progress curve plot.

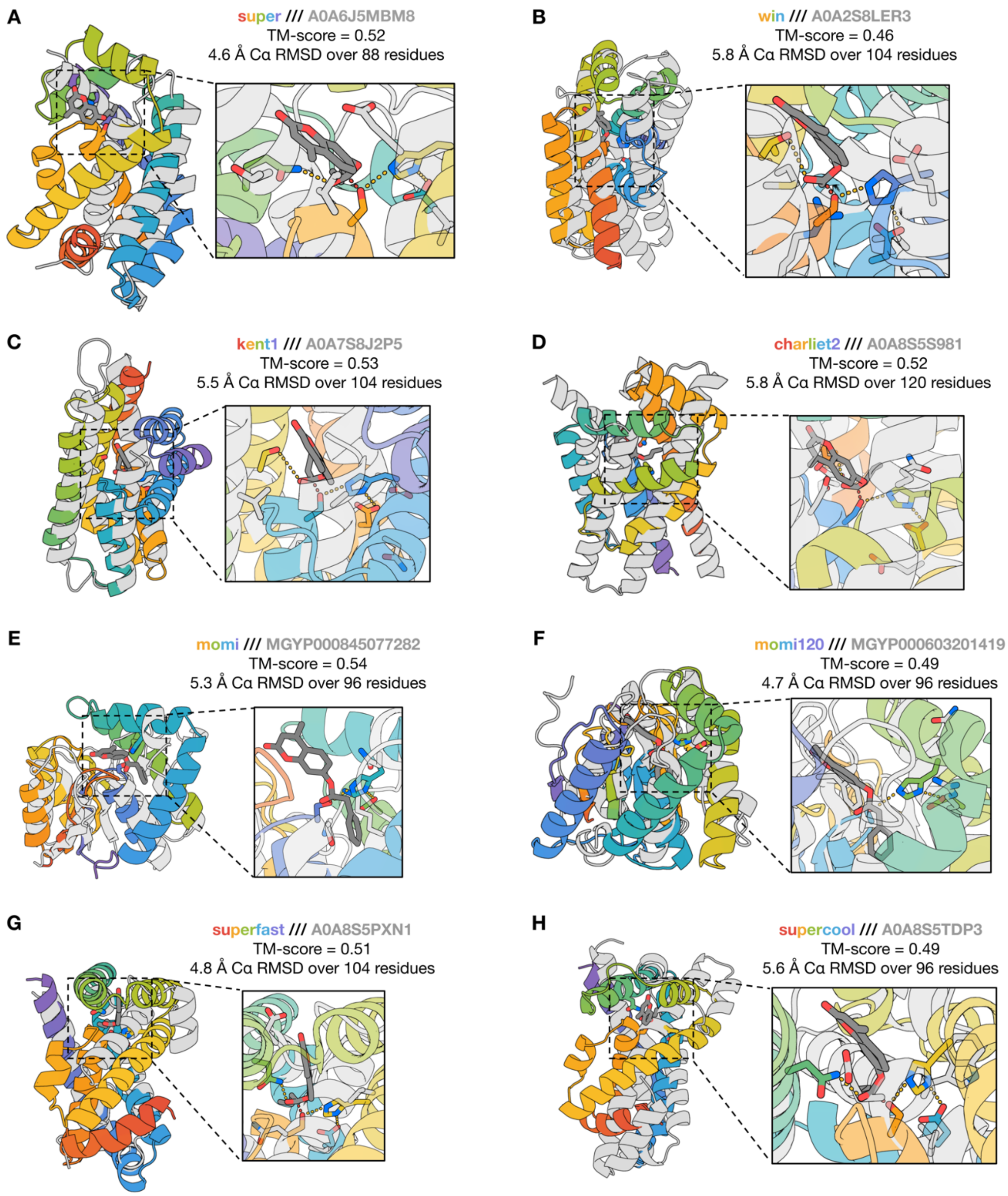


**Figure S10. Structural novelty of designed esterases.** Overlay of design models and most similar structures by TM-score from Foldseek searches for (**A**) **super**, (**B**) **win31**, (**C**) **kent1**, (**D**) **charliet2**, (**E**) **momi**, (**F**) **momi120**, (**G**) **superfast**, and (**H**) **supercool.**

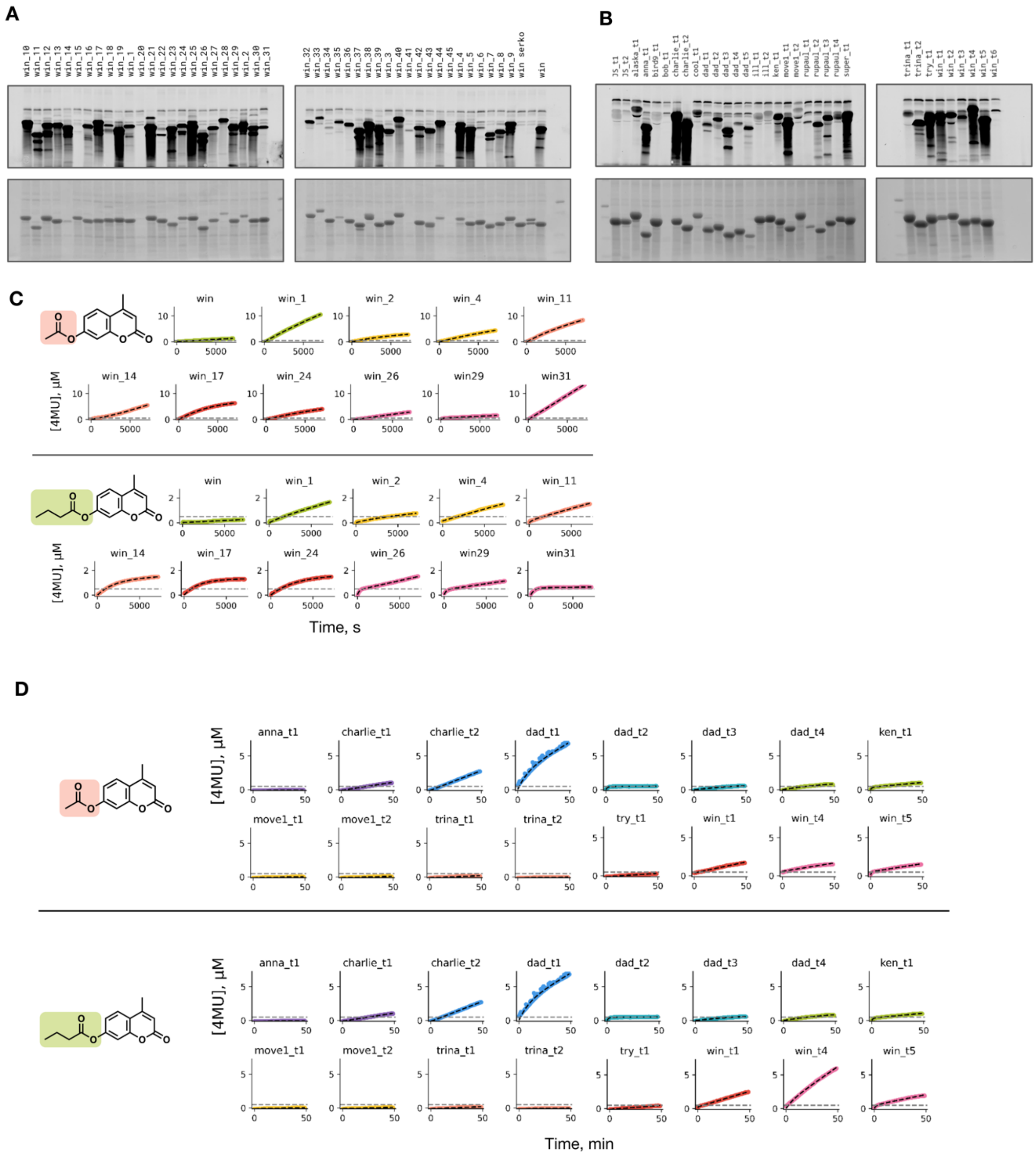


**Fig. S11. Screening and kinetic analysis of resampled designs.** In-gel fluorescence imaging after incubation of cell lysates of (A) **r3-win** redesigns and (B) round 3 redesigns with 1 µM FP-TAMRA. Progress curves of (C) **r3-win** redesigns and (D) round 3 redesigns incubated at 30°C with 100 µM 4MU-Ac and 4MU-Bu in 20 mM HEPES, 50 mM NaCl, pH 7.4, 5% DMSO assay buffer. Dashed line represents enzyme concentration (0.5 µM).

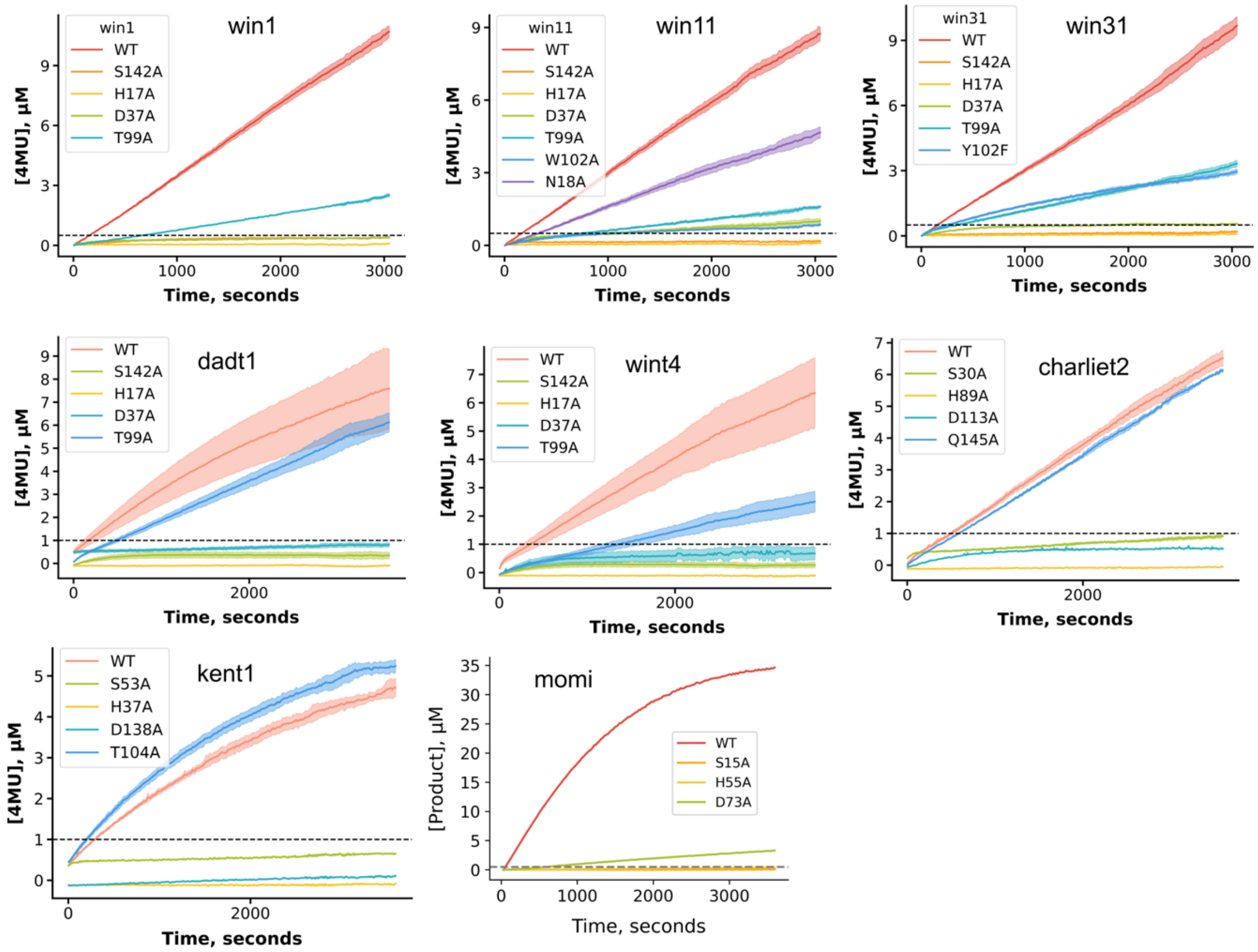


**Figure S12. Progress curves for active site residue mutants of designed serine hydrolases.** Reactions were performed at 30°C with 1 µM or 0.5 µM (**win1**, **win11**, **win31**) enzyme and 100 µM 4MU-Ac, 100 µM 4MU-Bu (**charliet2**, **kent1**), or 50 µM 4MU-PhAc (**momi**) in 20 mM HEPES, 50 mM NaCl, pH 7.4, 5% DMSO. Dashed lines indicate the enzyme concentration and shaded areas represent standard deviation of three technical replicates.

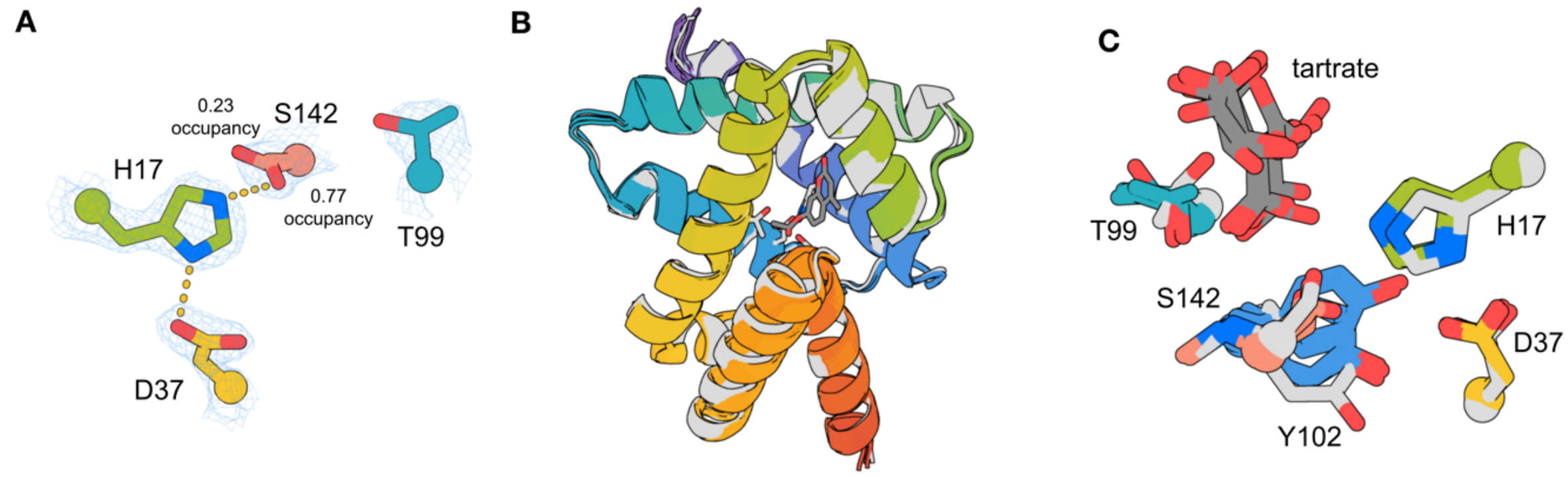


**Figure S13. Analysis of win1 and win31 crystal structures.** (**A**) The catalytic serine S142 in chain B of **win1** occupies two distinct rotameric states. 2Fo-Fc map in blue mesh shown at 1 σ. (**B**) Structural superposition of overall fold of all five chains in the asymmetric unit of **win31** (color) and **win31** design model (gray) (**C**) Active site zoom-in of structural superposition of the five chains in the **win31** asymmetric unit (color) overlaid with design model (gray).

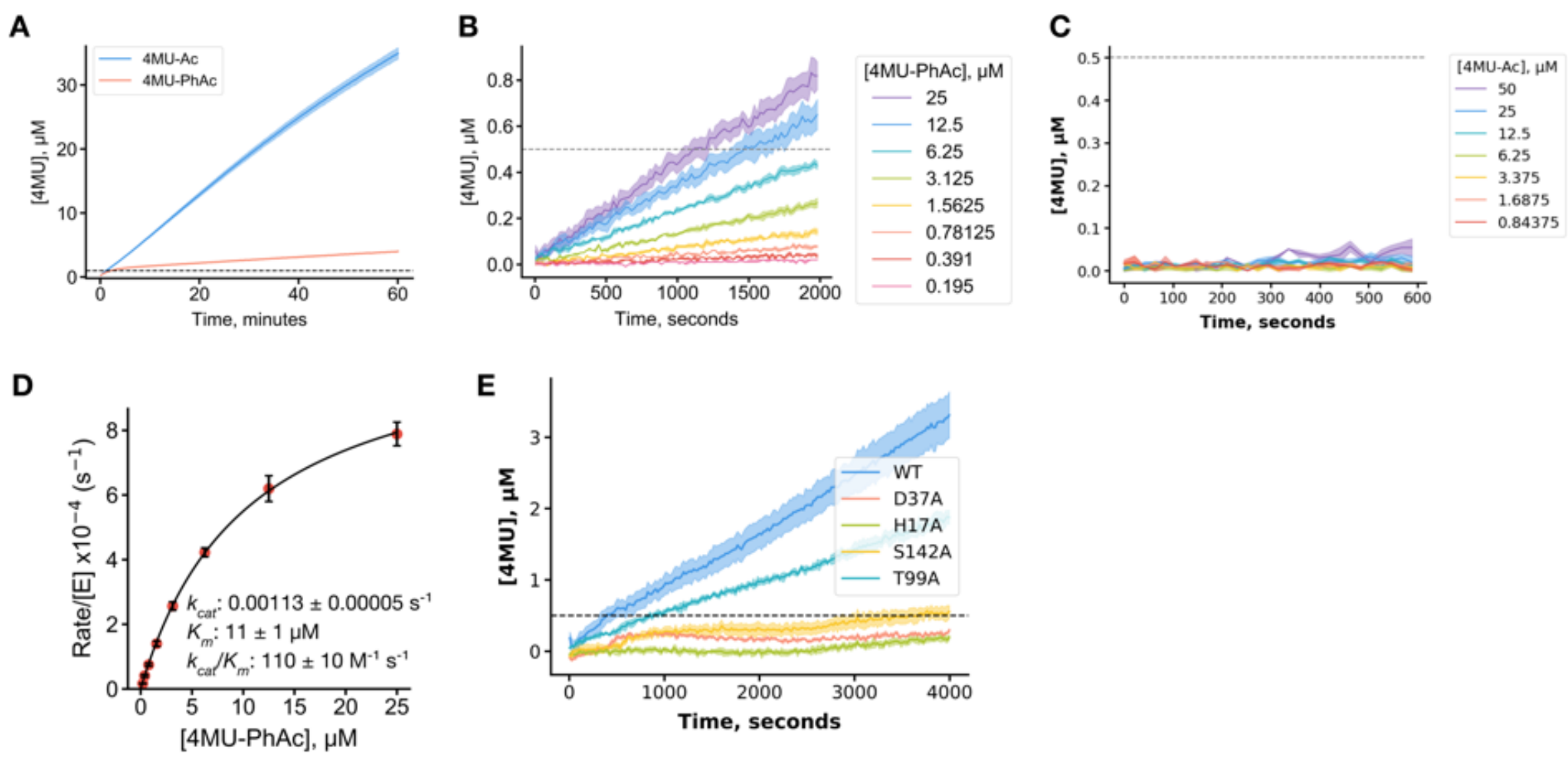


**Figure S14. Characterization of redesigns of win1 for larger 4MU-PhAc substrate.** (**A**) Progress curves of **win1** with 100 µM 4MU-Ac (blue) and 4MU-PhAc (red) indicate preference for 4MU-Ac. (**B**,**C**) Progress curves of **win1_b1** with 4MU-PhAc (**B**) and 4MU-Ac (**C**). (**D**) Michaelis-Menten plot of **win1_b1** with 4MU-PhAc. (**E**) Progress curves for catalytic residue knockout mutants of **win1_b1** with 100 µM 4MU-PhAc. Reactions were performed at 30°C in 20 mM HEPES, 50 mM NaCl, pH 7.4, 5% DMSO with 1 µM (**win1**) or 0.5 µM (**win1_b1**) enzyme. Dashed lines indicate the enzyme concentration and shaded areas represent standard deviation of three technical replicates.

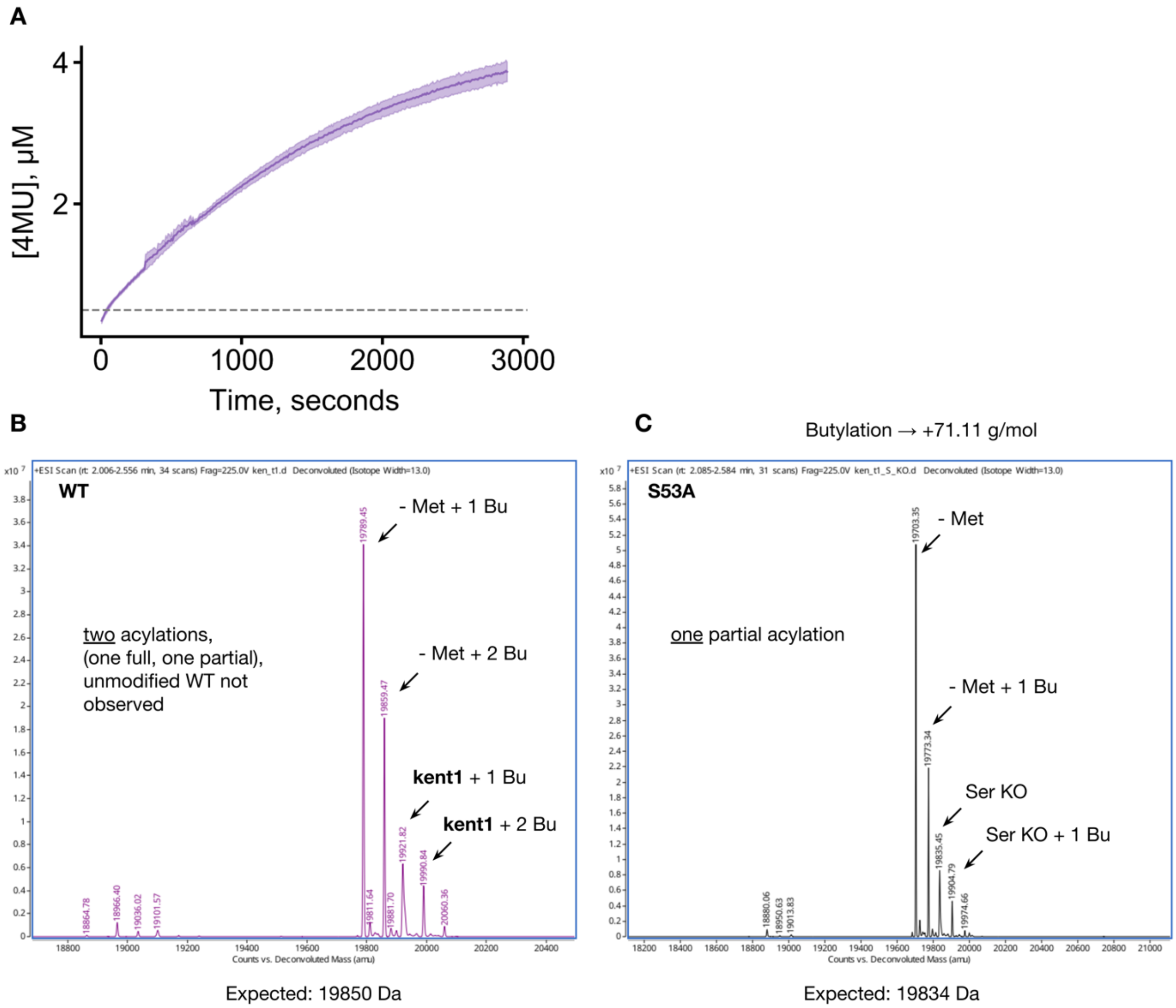


**Figure S15. Kinetic and mass spectrometric analysis of kent1 reveal inactivation over time and stable acylated species.** (**A**) Progress curve of 0.5 µM **kent1** incubated with 50 µM 4MU-Bu in 20 mM HEPES, 50 mM NaCl, pH 7.4, 5% DMSO assay buffer at 30°C. Dashed line represents the enzyme concentration and shaded area represents standard deviation of three technical replicates. (**B**) Mass spectra of WT and catalytic serine knockout (S53A) of 50 µM **kent1** after overnight incubation at room temperature with 100 µM 4MU-Bu.

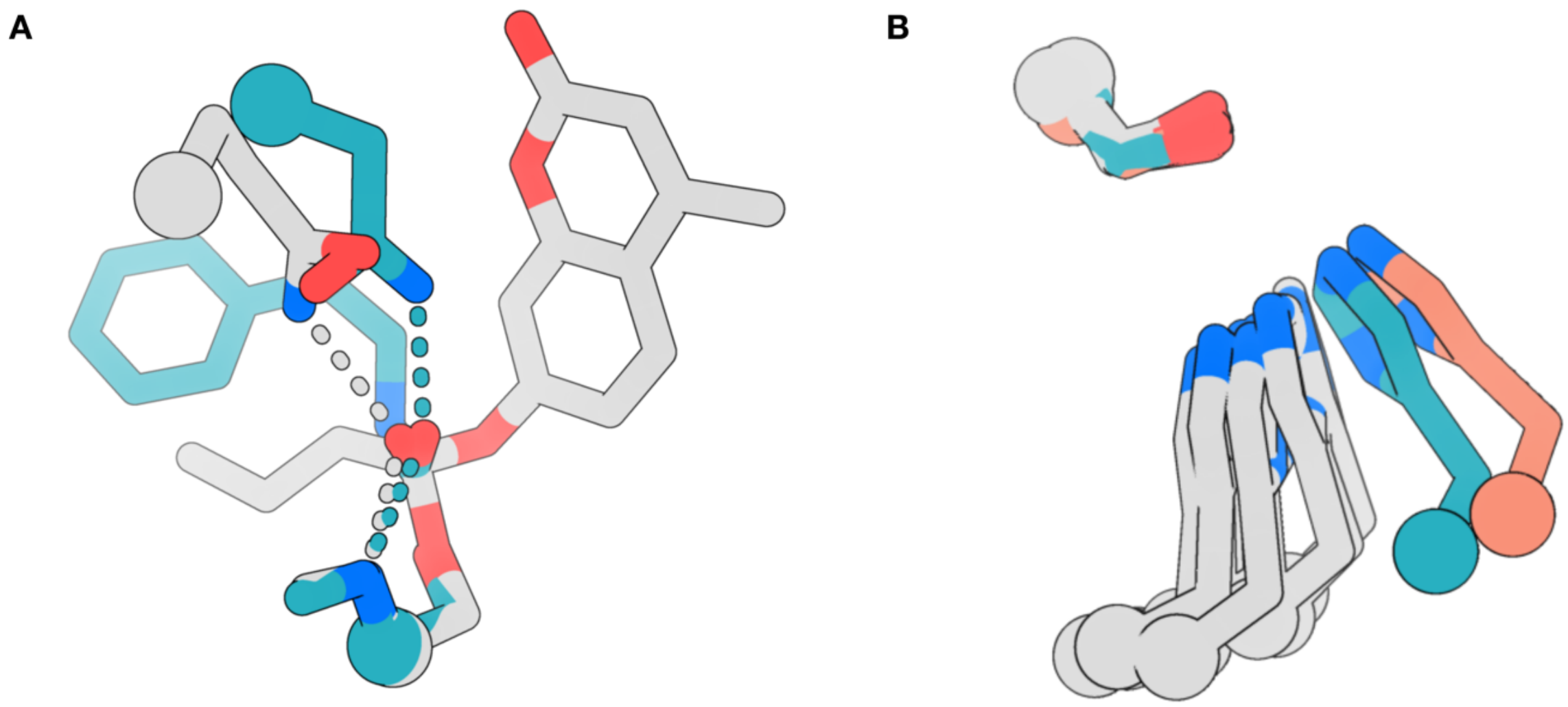


**Figure S16. Comparison of catalytic geometries in designed and native serine hydrolases.** (**A**) Structural alignment of **super** design model (gray) and crystal structure of subtilisin (teal) (PDB: 1scn) highlighting distinct oxyanion hole geometries of side chain glutamine (super) and asparagine (subtilisin) relative to the substrate carbonyl. **super's** oxyanion H-bond may stabilize the ground state and slow catalysis. (**B**) Comparison of His-Ser interactions found among native hydrolases (gray) and those in **super** (teal) and **win** (peach).

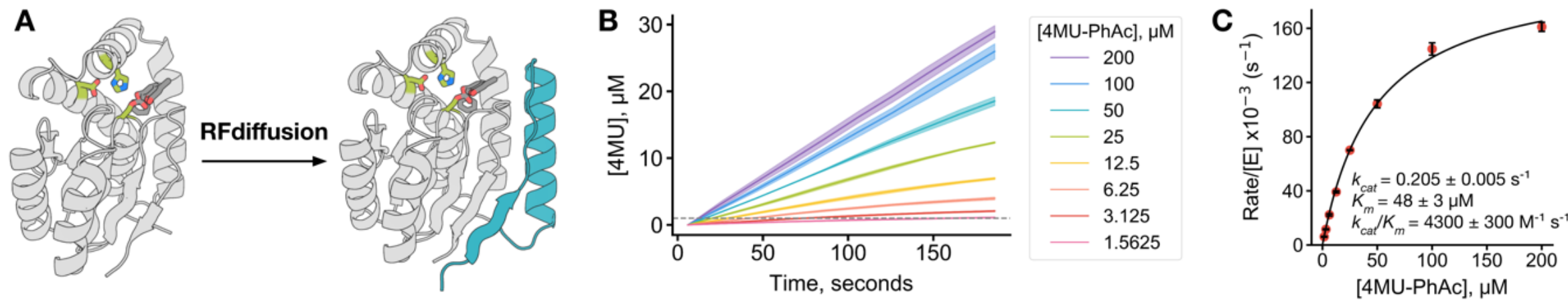


**Figure S17. Adding second-shell structural support to the active site of momi improves catalysis.** (**A**) Structural extension of N-terminus of **momi** with RFdiffusion to generate **momi120**. Original structure in gray, newly built region in teal. (**B**) Progress curves of **momi120** with increasing concentrations of 4MU-PhAc. Horizontal dashed lines indicate the enzyme concentration (1 μM) and shaded areas represent the standard deviation of three technical replicates. (**C**) Michaelis-Menten plot of **momi120** with 4MU-PhAc. Kinetic assays were performed in 20 mM HEPES, 50 mM NaCl, pH 7.4, 5% DMSO assay buffer at 30°C.

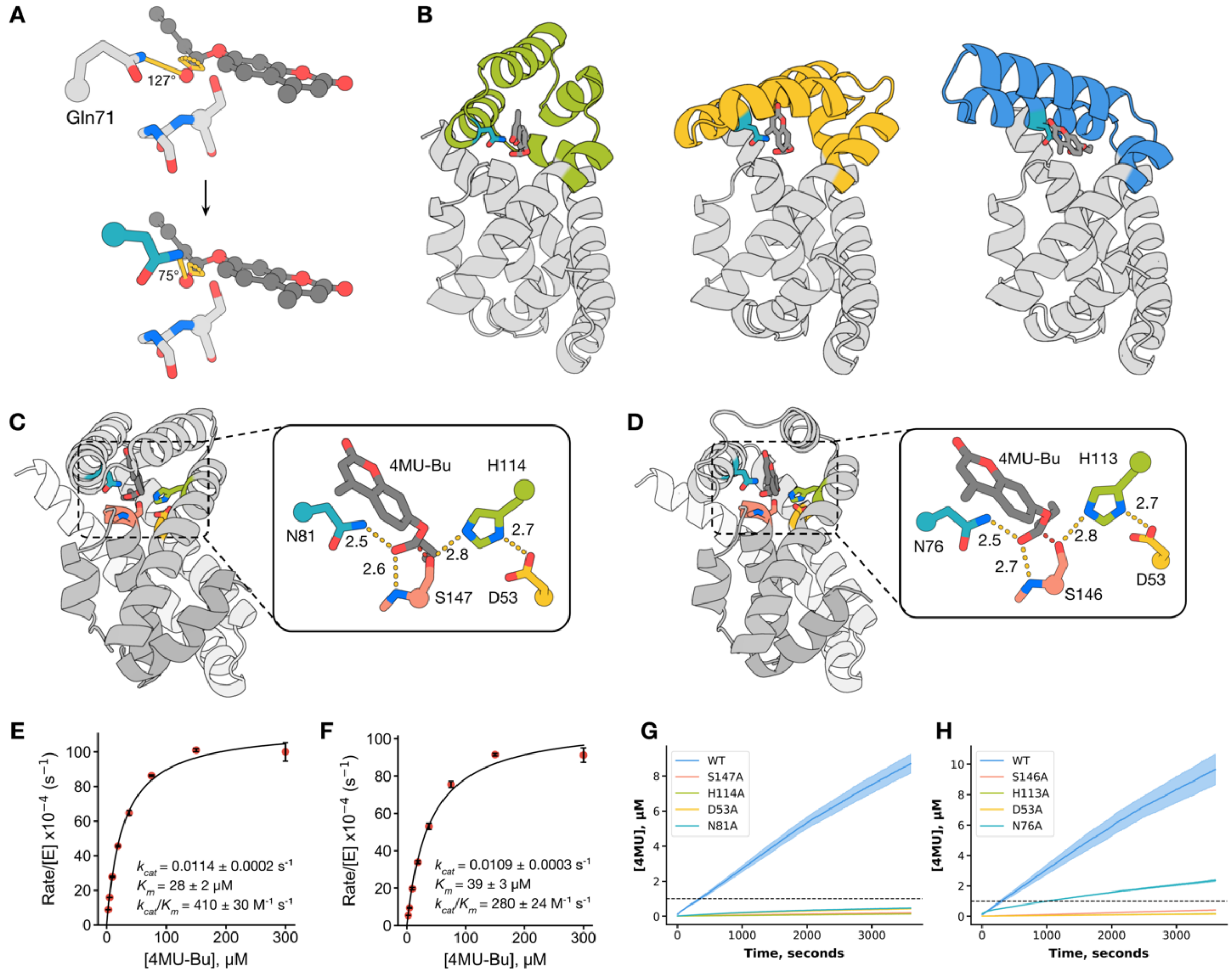


**Figure S18. Adjusting super oxyanion hole geometry with RFdiffusion improves catalysis.** (**A**) Repositioning of sidechain oxyanion hole Gln71 to a form a more out-of-plane H-bond with substrate carbonyl group (dihedral angle indicated in yellow between substrate ester and amino group shifted from 127 degrees in starting structure to 75 degrees). (**B**) Selected design models illustrating the diversity of the RFdiffusion-built region (green/yellow/blue) scaffolding the new sidechain oxyanion hole residue (teal). (**C**,**D**) Overall fold and active site zoom-in of **superfast** (C) and **supercool** (D) design model. (E,F) Michaelis-Menten plot of **superfast** (E) and **supercool** (F) with 4MU-Bu. (G,H) Progress curves of catalytic residue knockout mutants of **superfast** (G) and **supercool** (H). Dashed line represents the enzyme concentration (1 μM) and shaded area represents standard deviation of three replicates.

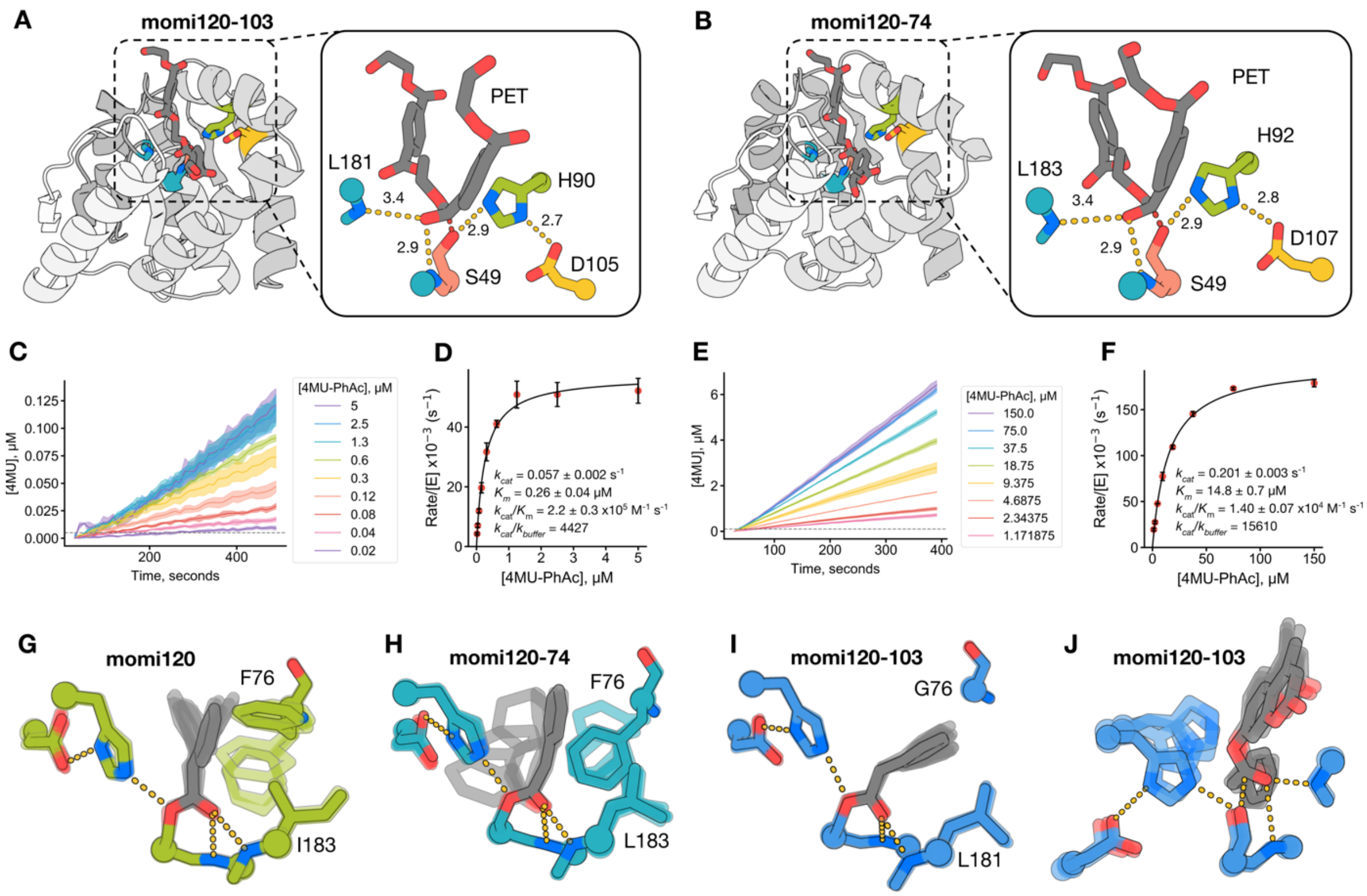


**Figure S19. Structural modeling and kinetic characterization of momi120 redesigns.** (A,B) Overall fold and active site zoom-in of momi120-103 (A) and momi120-74 (B) design models. (C,E) Progress curves of momi120-103 (C) and momi120-74 (E) incubated with 4MU-PhAc. (D,F) Michaelis-Menten plots of momi120-103 (D) and momi120-74 (F) with 4MU-PhAc. (G-I) PLACER ensembles of momi120 (G), momi120-74 (H), and momi120-103 (I) in the AEI state, highlighting active site differences involved in substrate binding at positions 76 and 181/183. (J) Superposition of five Chai-1 predictions of momi120-103 in complex with 4MU-PhAc.

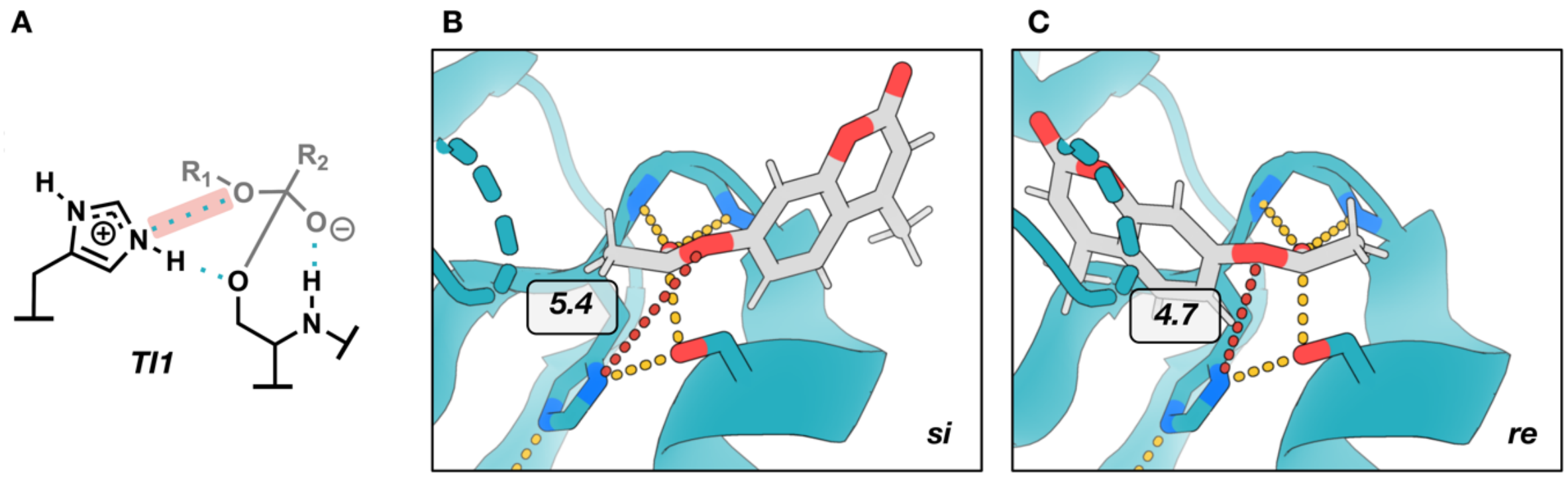


**Figure S20. Catalytically incompatible geometries in previous serine hydrolase design OSH55. (A)** Key interaction in tetrahedral intermediate 1 (TI1) between histidine Nε2 and substrate leaving group oxygen (red highlight). (**B, C**) Crystal structure of OSH55 design (E6D/L155R variant, PDB ID 4ess) shown in complex with computationally docked 4MU-Ac esterase substrate for both *re* and *si* faces of attack. The key histidine-leaving group interaction is depicted with red dashes. Distances in Å.

**Table S1. Kinetically or structurally analyzed designs from each round.**

| Design round | Design names |
|---|---|
| Round 0 | n8 |
| Round 1 | shh25 |
| Round 2 | rem6507 |
| Round 3 | super, win, josie |
| Round 4 | win1, win11, win31, dadt1 |
| Round 5 | charliet2, kent1 |
| Round 6 | win1_b1 |
| Round 7 | momi |
| Round 8 | momi120 |
| Round 9 | supercool, superfast |
| Round 10 | momi120_74, momi120_103 |

**Table S2. Data collection and refinement statistics.** Statistics for the highest resolution shell are shown in parentheses.

| | r0-n8 | r3-super | r3-win | r4-win1 | r4-win31 | r4-dadt1 |
|---|---|---|---|---|---|---|
| **PDB ID** | 9DED | 9DEE | 9DEF | 9DEG | 9DEH | 9MRB |
| **Wavelength** | 0.97648 | 0.92010 | 1.00002 | 1.00002 | 0.97936 | 0.97934 |
| **Resolution range** | 40.00 - 1.77 (1.81 - 1.77) | 32.12 - 1.21 (1.28 - 1.21) | 46.02 - 1.75 (1.78 - 1.75) | 43.27 - 2.11 (2.17 - 2.11) | 58.33 - 2.21 (2.33 - 2.21) | 51.19 - 2.001 (2.13 - 2.0) |
| **Space group** | P 1 21 1 | R 3 :H | P 21 2 21 | P 21 21 21 | P 21 21 21 | C 1 2 1 |
| **Unit cell** | 38.6, 31.6, 40.8; 90, 101.3, 90 | 68.4, 68.4, 76.4; 90, 90, 120 | 31.2, 46.0, 92.9; 90, 90, 90 | 35.8, 82.0, 101.9; 90, 90, 90 | 63.4, 107.0, 116.7; 90, 90, 90 | 42.4, 56.7, 51.6; 90, 97.23, 90 |
| **Total reflections** | 63121 (3594) | 2592198 (38409) | 153901 (8688) | 192423 (15480) | 457352 (66573) | 35618 (6028) |
| **Unique reflections** | 9440 (526) | 40645 (5957) | 14105 (749) | 17989 (1414) | 40598 (5849) | 8217 (1357) |
| **Multiplicity** | 6.7 (6.8) | 6.4 (6.4) | 10.9 (11.6) | 10.7 (10.9) | 11.3 (11.4) | 4.3 (4.4) |
| **Completeness (%)** | 98.8 (99.3) | 99.86 (100.00) | 99.80 (99.60) | 99.9 (99.9) | 100.00 (100.00) | 98.77 (98.62) |
| **Mean I/sigma(I)** | 11.4 (2.4) | 12.6 (1.1) | 14.0 (1.9) | 10.3 (2.4) | 6.4 (0.70) | 4.04 (0.92) |
| **Wilson B-factor** | 22 | 21 | 27 | 28 | 39 | 29 |
| **R-merge** | 0.090 (0.831) | 0.048 (1.347) | 0.084 (1.274) | 0.151 (0.958) | 0.284 (3.183) | 0.185 (1.494) |
| **R-pim** | 0.041 (0.368) | 0.022 (0.639) | 0.028 (0.402) | 0.050 (0.314) | 0.090 (1.024) | 0.099 (0.787) |
| **CC1/2** | 0.997 (0.798) | 0.999 (0.471) | 0.998 (0.752) | 0.999 (0.918) | 0.977 (0.424) | 0.992 (0.566) |
| **Reflections used in refinement** | 9422 (1332) | 40645 (2937) | 14057 (1366) | 17872 (1321) | 40457 (2855) | 8173 (1355) |
| **Reflections used for R-free** | 935 (138) | 2012 (150) | 1406 (137) | 1788 (131) | 2004 (138) | 818 (136) |
| **R-work** | 0.1896 (0.2479) | 0.2057 (0.3380) | 0.2361 (0.3217) | 0.2039 (0.2557) | 0.2381 (0.3796) | 0.2131 (0.2667) |
| **R-free** | 0.2216 (0.2904) | 0.2414 (0.3917) | 0.2726 (0.3556) | 0.2590 (0.3229) | 0.2697 (0.3863) | 0.2575 (0.3123) |
| **Number of non-hydrogen atoms** | 1044 | 1451 | 1284 | 2700 | 6346 | 1181 |
| **macromolecules** | 969 | 1292 | 1215 | 2535 | 6204 | 1131 |
| **ligands** | 0 | 0 | 5 | 6 | 83 | 13 |
| **solvent** | 75 | 159 | 64 | 159 | 59 | 37 |

| | | | | | | |
|---|---|---|---|---|---|---|
| **Protein residues** | 114 | 160 | 151 | 314 | 790 | 141 |
| **RMS(bonds)** | 0.019 | 0.006 | 0.007 | 0.011 | 0.008 | 0.002 |
| **RMS(angles)** | 1.62 | 0.60 | 0.81 | 1.03 | 0.75 | 0.40 |
| **Ramachandran favored (%)** | 98.21 | 99.37 | 97.99 | 99.68 | 99.36 | 100.00 |
| **Ramachandran allowed (%)** | 1.79 | 0.63 | 2.01 | 0.32 | 0.64 | 0 |
| **Ramachandran outliers (%)** | 0.00 | 0.00 | 0.00 | 0.00 | 0.00 | 0 |
| **Average B-factor** | 26 | 27 | 40 | 33 | 49 | 31 |
| **macromolecules** | 26 | 26 | 40 | 33 | 49 | 31 |
| **ligands** | | | 34 | 51 | 56 | 44 |
| **solvent** | 315 | 35 | 35 | 36 | 40 | 34 |

**Table S3. Template indicator, timestep and structural features input to both the diffusion and refinement models of CA RFdiffusion.** Template #1 is the self-conditioning template, exactly as described in *(36)*. For the new per token “is motif” indicator, this template receives a null value of -1 for all tokens, while still containing the encoding of the current discrete time step 1-T/t. The pairwise template features for template #1 contain the self-conditioning information (the distance/angle encoding of the previous prediction of $X_0$), and a null -1 indicator feature for all pairs. Template #2 contains null information (i.e., -1) for all indicator and timestep features, but the pairwise structure input contains the distance/angle encoding of the current noisy $X_t$ structure being input to the network in 3D. Template #3 contains information about the perfect motif geometry and primary sequence location, to be scaffolded by the network. This template is the only one of the three which uses the “is motif” feature (0 if non-motif, 1 if motif token) and the “pair is constrained” indicator (1 if the pair is constrained, else 0).

| | **Per-token (1D) info** | **Per-token-pair (2D) info** |
|---|---|---|
| **Template #1 -** Self-conditioning | **Timestep feature:** 1-T/t<br>**Is motif feature:** -1 | **Structural data:** Previous prediction of $X_0$<br>Pair constraint indicator: -1 |
| **Template #2 -** Current $X_t$ input | **Timestep feature:** -1<br>**Is motif feature:** -1 | **Structural data:** Current $X_t$ structure input<br>Pair constraint indicator: -1 |
| **Template #3 -** Perfect motif info | **Timestep feature:** -1<br>**Is motif feature:** 0 for False, 1 for True | **Structural data:** Perfect motif geometry<br>**Pair constraint indicator:** 1 if token pair is geometrically constrained, else 0. |

**Table S4. Training dataset and masking strategy probabilities for diffusion model training.** For protein-only monomers from the PDB, which is chosen 60% of the time, there are three possible masking strategies. (A) A "chunked" diffusion mask, in which 1-8 discontiguous fragments are designated as motif components. Each pair of discontiguous fragments has some nonzero probability of being unconstrained with respect to each other (see Algorithm 1 for details). (B) Multiple "triple contacts" are chosen, in which three residues that are close in spatial proximity but far from each other in primary sequence are selected (see Algorithm 2 for details). (C) Unconditional, in which there are no motifs and the task is to denoise the full structure. For the second dataset, which is PDB monomers in complex with small molecule ligands, there is a single motif masking strategy used every time. In this "small molecule contact mask" generation, the structure of the ligand is revealed in the template, along with either 0, 1, or 2 additional discontiguous protein fragments in close proximity to the ligand (see Algorithm 3 for details).

| **Dataset probabilities** | **Masking probabilities** |
|---|---|
| Dataset #1: PDB monomers - 60% probability | (A) "Chunked diffusion mask" - 20%<br>(B) Multi triple contact - 50%<br>(C) Unconditional - 30% |
| Dataset #2: PDB monomers in complex with ligands - 40% probability | (A) Small molecule contact mask - 100% |

**Table S5. Training hyperparameters for CA RFdiffusion (diffusion model and refinement model).**

| Parameter | Description | Value |
|---|---|---|
| $\beta_0$ | Variance at time t=0 | 0.01 |
| $\beta_T$ | Variance at time t=T=200 | 0.07 |
| T | Total number of timesteps in noising process | 200 |
| Variance schedule type | NA | Linear |
| $W_{disp}$ | CA displacement loss weight | 0.5 |
| $W_{dist\text{-}ang}$ | Distogram and anglogram cross entropy loss weight | 0.05 |
| $W_{FAPE,prot}$ | Intra-protein fragment FAPE loss weight | 10.0 |
| $W_{FAPE,prot\text{-}sm}$ | Inter protein-ligand FAPE loss weight | 10.0 |
| $W_{FAPE,sm}$ | Intra ligand FAPE loss weight | 10.0 |
| $W_{FAPE,prot(non\text{-}motif)}$ | (Refinement model only) Non-motif protein FAPE loss weight | 10.0 |
| $W_{pLDDT}$ | (Refinement model only) pLDDT loss weight | 0.1 |
| $A_{FAPE,prot}$ | Normalizing constant for protein motif FAPE | 5.0 |
| $A_{FAPE,sm}$ | …for intra-ligand FAPE | 4.0 |
| $A_{FAPE,prot\text{-}sm}$ | …for inter protein-ligand FAPE | 10 |

| | | |
|---|---|---|
| $A_{FAPE,prot(non\text{-}motif)}$ | …for non-motif FAPE. | 10 |
| $D_{clamp,prot}$ | Clamp upper bound for protein motif FAPE | 5 |
| $D_{clamp,sm}$ | …for intra-ligand FAPE | 4.0 |
| $D_{clamp,prot\text{-}sm}$ | …for inter protein-ligand FAPE | 10 |
| $D_{clamp,prot(non\text{-}motif)}$ | …for non-motif FAPE | 10 |
| Batch size | Effective batch size on 8GPUs | 16 |
| Learning rate | NA | 0.0005 |
| p_show_motif_seq | Probability of showing the amino acid identity of non-ligand motif tokens | 0.65 |
| Diffusion coordinate scaling | Scaling factor applied to coordinates before the forward process, then inverted and applied after the forward process. | 0.25 |
| Refinement model 3D Gaussian variance | Variance for noising CA atoms in structures. | 1.0 for epochs 1-4, 1.5 for epochs 5-7. |
| Epoch size | Number of examples per training epoch | 25600 |

**Table S6. Dataset proportions, mask sampling proportions, and 3D Gaussian noise variance for the two stage refinement model training procedure.**

| Refinement model training stage | Dataset probabilities and mask probabilities | 3D Gaussian noise variance |
|---|---|---|
| 1 | Datasets: PDB monomer - 100%<br><br>MasksTraining: “Chunked diffusion mask” - 20%<br>Multi triple contact - 50% | 1.0 |
| 2 | Datasets: PDB monomer - 40%<br>PDB monomer + ligand - 60%<br><br>Masks (for PDB monomer set):<br>“Chunked diffusion mask” - 20%<br>Multi triple contact - 50%<br>Unconditional - 30%<br><br>Masks (for PDB monomer + ligand set):<br>Small molecule contact mask - 100% | 1.5 |

**Algorithm 1. Small molecule contact mask generation.** This function defines how 1D and 2D motif masks were generated for protein-ligand complex examples.

```
def _get_diffusion_mask_chunked(xyz, prop_low, prop_high, max_motif_chunks=6):
   """
   Masking strategy that creates discontiguous motifs, possibly unconstrained w.r.t each other.
Parameters:
xyz (torch.tensor, required): (L,14,3) tensor of coordinates
prop_low (float, required): lower bound on fraction of protein to mask
prop_high (float, required): upper bound on fraction of proteins to mask
   """
   chunks, chunk_is_motif, motif_ids, ij, ij_can_see = get_chunked_mask(xyz, prop_low, prop_high,
max_motif_chunks)
   chunk_starts = np.cumsum([0] + chunks[:-1])
   chunk_ends   = np.cumsum(chunks)
   # make 1D array designating which chunks are motif
   L = xyz.shape[0]
   mask = torch.zeros(L, L)
   is_motif = torch.zeros(L)
   for i in range(len(chunks)):
       is_motif[chunk_starts[i]:chunk_ends[i]] = chunk_is_motif[i]
   # 2D array designating which chunks can see each other
   for i in range(len(chunks)):
       for j in range(len(chunks)):
           i_is_motif = chunk_is_motif[i]
           j_is_motif = chunk_is_motif[j]
           if (i_is_motif and j_is_motif): # both are motif, so possibly reveal info
               ID_i = motif_ids[i]
               ID_j = motif_ids[j]
               assert ID_i != -1 and ID_j != -1, 'both motif but one has no ID'
               # always reveal self vs self
               if i == j:
                   mask[chunk_starts[i]:chunk_ends[i], chunk_starts[j]:chunk_ends[j]] = 1
               else:
                   # find out of this (i,j) are allowed to see each other
                   ix = tuple(sorted([ID_i, ID_j]))
                   can_see = ij_can_see[ij.index(ix)]
                   if can_see:
                       mask[chunk_starts[i]:chunk_ends[i], chunk_starts[j]:chunk_ends[j]] = 1
   return mask.bool(), is_motif.bool()


def get_chunked_mask(xyz, low_prop, high_prop, max_motif_chunks=8):
   """
   Produces a mask of discontiguous protein chunks that are revealed.
   Also produces a tensor indicating which chunks are given relative geom. info
   Parameters:
   -----------
   xyz (torch.tensor): (L, 14, 3) tensor of atomic coordinates
   low_prop (float): lower bound on proportion of protein that is masked
   high_prop (float): upper bound on proportion of protein that is masked
   """
   L = xyz.shape[0]
   # decide number of chunks
   n_motif_chunks = random.randint(2, max_motif_chunks)
   # decide what proportion of the protein is masked
   # prop cannot result in n_unmasked < n_motif_chunks --> clamp high prop
   max_prop = 1-(n_motif_chunks+1)/L       # add +1 to be safe
   high_prop = min(high_prop, max_prop)
   prop = random.uniform(low_prop, high_prop)
   n_masked   = int(L * prop)
   n_unmasked = L - n_masked
   # decide the length of each chunk by randomly sampling
   # positions to cut a line with n_chunks - 1 cuts
   cuts     = sorted(random.sample(range(1, n_unmasked), n_motif_chunks - 1))
```

```
    lengths = [cuts[0]] + [cuts[i] - cuts[i-1] for i in range(1, len(cuts))] + [n_unmasked -
cuts[-1]]
    # decide which chunks are given relative geom. info
    # walk over all unique pairs
    motif_pairs = list(itertools.combinations(range(n_motif_chunks), 2))
    # 33% chance that a pair can see each other
    pairs_can_see = [random.choice([True, False, False]) for _ in range(len(motif_pairs))]
    # decide location of chunks within the protein
    # (1) decide order
    random.shuffle(lengths)
    # (2) split available space remaining into other chunks between
    #     the chunks that have been assigned a length
    ngap_low  = len(lengths) - 1
    ngap_high = ngap_low + 1
    ngap = random.randint(ngap_low, ngap_high)
    if ngap == (ngap_low): # there's no cterm/nterm gaps
        Nterm_gap = False
        Cterm_gap = False
    elif ngap == (ngap_low + 1): # there's either a cterm or nterm gap
        Nterm_gap = random.choice([True, False])
        Cterm_gap = not Nterm_gap
    else: # there's both a cterm and nterm gap
        Nterm_gap = True
        Cterm_gap = True
    gaps = sample_gaps(ngap, n_masked) # gaps between unmasked chunks
    random.shuffle(gaps)
    chunks = []
    is_motif = []
    motif_ids = []
    cur_motif_id = 0
    if Nterm_gap:
        chunks.append(gaps.pop())
        is_motif.append(False)
        motif_ids.append(-1)
    for i in range(len(lengths)):
        chunks.append(lengths[i])
        is_motif.append(True)
        motif_ids.append(cur_motif_id)
        cur_motif_id += 1
        if len(gaps) > 1:                          # more to spare
            chunks.append(gaps.pop())
            is_motif.append(False)
            motif_ids.append(-1)
        elif len(gaps) == 1 and not Cterm_gap:   # only one left, but no Cterm gap
            chunks.append(gaps.pop())
            is_motif.append(False)
            motif_ids.append(-1)
        else:
            pass # no more to spare
    if Cterm_gap:
        assert len(gaps) == 1
        chunks.append(gaps.pop())
        is_motif.append(False)
        motif_ids.append(-1)
    assert sum(chunks) == L, f'chunks sum to {sum(chunks)} but should sum to {L}'
    return chunks, is_motif, motif_ids, motif_pairs, pairs_can_see
def sample_gaps(n, M):
    """
    Samples n chunks that sum to M.
              https://stackoverflow.com/questions/2640053/getting-n-random-numbers-whose-sum-is-
m/2640079#2640079
    Parameters:
n (int, required): number of chunks
M (int, required): number that the chunk lengths should sum to
    """
    nums = np.random.dirichlet(np.ones(n))*M
    # now round to nearest integer, conserving the total sum
    rounded = []
    round_up = True
    for i in range(len(nums)):
        if round_up:
```

```
            rounded.append(np.ceil(nums[i]))
            round_up = False
        else:
            rounded.append(np.floor(nums[i]))
            round_up = True
    # ensure all > 0
    for i in range(len(rounded)):
        if rounded[i] < 1:
            rounded[i] = 1
    while sum(rounded) > M:
        ix = np.random.randint(0, len(rounded))
        if rounded[ix] < 2: # must be at least 1
            continue
        rounded[ix] -= 1
    while sum(rounded) != M:
        ix = np.random.randint(0, len(rounded))
        rounded[ix] += 1
    assert all([x >= 1 for x in rounded])
    return [int(x) for x in rounded]
```

**Algorithm 2. Multi triple contact.** These functions define how to retrieve a "multi triple contact" motif mask.

```
def _get_multi_triple_contact_3template(xyz,
                                        low_prop,
                                        high_prop,
                                        max_triples=2,
                                        xyz_less_than=6,
                                        seq_dist_greater_than=10,
                                        len_low=1,
                                        len_high=7,
                                        force_triples=None):
   """
   Gets 2d mask + 1d is motif for multiple triple contacts.
   Parameters:
       xyz (torch.tensor, required): (L, 14, 3) atomic coordinates
       low_prop (float, required): lower bound for proportion of protein to mask
       high_prop (float, required): upper bound for proportion of protein to mask
       max_triples (int, optional): maximum number of triples to find. Default is 2.
       xyz_less_than (int, optional): maximum distance between atoms to consider a contact.
Default is 6.
       seq_dist_greater_than (int, optional): minimum sequence distance between atoms to consider
a contact. Default is 10.
       len_low (int, optional): minimum length of motif chunk. Default is 1.
       len_high (int, optional): maximum length of motif chunk. Default is 7.
       force_triples (int, optional): force the number of triples to be this number. Default is
None.
   """
   contacts = get_contacts(xyz, xyz_less_than, seq_dist_greater_than)
   if not contacts.any():
       return _get_diffusion_mask_chunked(xyz, low_prop, high_prop, max_motif_chunks=6)
   is_motif_stack = []
   mask_2d_stack = []
   if force_triples is None:
       n_triples = random.randint(1, max_triples)
   else:
       n_triples = force_triples

   for i in range(n_triples):
       indices = find_third_contact(contacts)

       if (indices is None):
           if i == 0:
               # we found no triples at all, so just return a simple chunked diffusion mask
               return _get_diffusion_mask_chunked(xyz, low_prop, high_prop, max_motif_chunks=6)
           else:
               # we found i triples but couldn't find i+1 --> regenerate and return the i triples
               return _get_multi_triple_contact_3template(xyz, low_prop, high_prop,
force_triples=i)
       L = xyz.shape[0]
       # 1d tensor describing which residues are motif
       tmp_is_motif = sample_around_contact(L, indices, len_low, len_high)
       # now get the 2d tensor describing which residues can see each other
       # For these, all motif chunks can see each other
       tmp_mask_2d = tmp_is_motif[:, None] * tmp_is_motif[None, :]
       is_motif_stack.append(tmp_is_motif)
       mask_2d_stack.append(tmp_mask_2d)
   is_motif = torch.stack(is_motif_stack, dim=0).bool()
   mask_2d = torch.stack(mask_2d_stack, dim=0).bool()
   is_motif = torch.any(is_motif, dim=0)
   mask_2d = torch.any(mask_2d, dim=0)
   return mask_2d, is_motif
def sample_around_contact(L, indices, len_low, len_high):
   """
   Given a list of indices, sample a revealed motif around each index.
   Parameters:
       L (int, required): length of protein
       indices (list, required): list of indices around which to sample motif
       len_low (int, optional): minimum length of motif.
       len_high (int, optional): maximum length of motif.
```

```
    """
    diffusion_mask = torch.zeros(L).bool()
    for anchor in indices:
        mask_length = int(np.floor(random.uniform(len_low, len_high)))
        l = anchor - mask_length // 2
        r = anchor + (mask_length - mask_length//2)
        l = max(0, l)
        r = min(r, L)
        diffusion_mask[l:r] = True
    return diffusion_mask
def get_Cb(xyz):
    """
    Compute Cb given N,Ca,C
    Parameters:
        xyz (torch.tensor, required): shape (batch, L, 3) Cartesian coordinates of atoms
    """
    N  = xyz[...,0,:]
    Ca = xyz[...,1,:]
    C  = xyz[...,2,:]
    b = Ca - N
    c = C - Ca
    a = torch.cross(b, c, dim=-1)
    return -0.58273431*a + 0.56802827*b - 0.54067466*c + Ca
def get_pair_dist(a, b):
    """
    calculate pair distances between two sets of points

    Parameters:
        a,b (torch.tensor, required): shape (batch, L, 3) Cartesian coordinates of atoms
    """
    dist = torch.cdist(a, b, p=2)
    return dist
def get_cb_distogram(xyz):
    Cb = get_Cb(xyz)
    dist = get_pair_dist(Cb, Cb)
    return dist
def get_contacts(xyz, xyz_less_than=5, seq_dist_greater_than=10):
    """
    Computes a 2D boolean mask of contacting residues. True if i,j are contacting, False
otherwise.
    """
    L = xyz.shape[0]
    dist = get_cb_distogram(xyz)
    is_close_xyz = dist < xyz_less_than
    idx = torch.ones_like(dist).nonzero()
    seq_dist = torch.abs(torch.arange(L)[None] - torch.arange(L)[:,None])
    is_far_seq = torch.abs(seq_dist) > seq_dist_greater_than
    contacts = is_far_seq * is_close_xyz
    return contacts
def find_third_contact(contacts):
    """
    Finds a third contact for a pair of contacting residues
    Parameters:
        contacts (torch.tensor, required): (L, L) 2d mask of contacts between residues.
    """
    contact_idxs = contacts.nonzero()
    contact_idxs = contact_idxs[torch.randperm(len(contact_idxs))]
    for i,j in contact_idxs:
        if j < i:
            continue
        K = (contacts[i,:] * contacts[j,:]).nonzero()
        if len(K):
            K = K[torch.randperm(len(K))]
            for k in K:
                return torch.tensor([i,j,k])
    return None
```

**Algorithm 3. Small molecule contact mask generation.** The following function defines how 1D and 2D motif masks were generated for protein-ligand complex examples.

```
def _get_sm_contact_3template(xyz,
                              is_sm,
                              contact_cut=8,
                              chunk_size_min=1,
                              chunk_size_max=7,
                              min_seq_dist=9):
   """
   Produces mask2d and is_motif for small molecule, possibly with contacting protein chunks 
revealed.
   Parameters:
       xyz (torch.Tensor): 3D coordinates of protein and small molecule (L, 14, 3)
       is_sm (torch.Tensor): binary tensor indicating which tokens are small molecule atoms (L,)
       contact_cut (float): distance cutoff for contact between protein and small molecule
       chunk_size_min (int): minimum size of revealed chunk
       chunk_size_max (int): maximum size of revealed chunk
       min_seq_dist (int): minimum sequence distance between revealed chunks
   """
   assert len(xyz.shape) == 3
   ca = xyz[~is_sm, 1,:]
   if ca.shape[0] == 0:
       sm_only = True
   else:
       sm_only = False
   sm_xyz  = xyz[is_sm, 1,:]
   dmap = torch.cdist(ca, sm_xyz)
   dmap = dmap < contact_cut
   protein_is_contacting = dmap.any(dim=-1) # which CA's are contacting sm
   where_is_contacting = protein_is_contacting.nonzero().squeeze()
   n_chunk_revealed = random.randint(0,4)
   if (n_chunk_revealed == 0) or (sm_only):
       is_motif = is_sm.clone()
       is_motif_2d = is_motif[:, None] * is_motif[None, :]
       return is_motif_2d, is_motif
   else:
       is_motif = is_sm.clone()
       cur_min_seq_dist = min_seq_dist # could possibly increment this if needed
       for i in range(n_chunk_revealed):
           chunk_size = torch.randint(chunk_size_min, chunk_size_max, size=(1,)).item()
           if len(where_is_contacting.shape) == 0:
               # ensures where_is_contacting is a 1d tensor
               where_is_contacting = where_is_contacting.unsqueeze(0)
           if (where_is_contacting.shape[0] == 0) and (i == 0):
               # no contacts, so sm is only motif
               is_motif = is_sm.clone()
               is_motif_2d = is_motif[:, None] * is_motif[None, :]
               return is_motif_2d, is_motif
           p = torch.ones_like(where_is_contacting)/len(where_is_contacting)
           chosen_idx = p.multinomial(num_samples=1, replacement=False)
           chosen_idx = chosen_idx.item()
           chosen_idx = where_is_contacting[chosen_idx]
           # find min and max indices for revealed chunk
           min_index = max(0, chosen_idx - chunk_size//2)
           max_index = min(protein_is_contacting.numel(), 1+chosen_idx + chunk_size//2)
           # reveal chunk
           is_motif[min_index:max_index] = True
           # update where_is_contacting
           start = max(0,min_index-cur_min_seq_dist)
           end = min(protein_is_contacting.numel(), max_index+cur_min_seq_dist)
           protein_is_contacting[start:end] = False # remove this option from where_is_contacting
           where_is_contacting = protein_is_contacting.nonzero().squeeze()
           if protein_is_contacting.sum() == 0:
               break # can't make any more chunks
       is_motif_2d = is_motif[:, None] * is_motif[None, :] # all tokens can "see" each other
       return is_motif_2d, is_motif
```
